# Treatment recommendations based on network meta-analysis: Rules for risk-averse decision-makers

**DOI:** 10.1017/rsm.2025.17

**Published:** 2025-04-24

**Authors:** A. E. Ades, Annabel L. Davies, David M. Phillippo, Hugo Pedder, Howard Thom, Beatrice Downing, Deborah M. Caldwell, Nicky J. Welton

**Affiliations:** Population Health Sciences, Bristol University Medical School, Bristol, UK

**Keywords:** decision-making, expected value, GRADE, loss-adjustment, network meta-analysis, treatment ranking

## Abstract

The treatment recommendation based on a network meta-analysis (NMA) is usually the single treatment with the highest expected value (EV) on an evaluative function. We explore approaches that recommend multiple treatments and that penalise uncertainty, making them suitable for risk-averse decision-makers. We introduce loss-adjusted EV (LaEV) and compare it to GRADE and three probability-based rankings. We define properties of a valid ranking under uncertainty and other desirable properties of ranking systems. A two-stage process is proposed: the first identifies treatments superior to the reference treatment; the second identifies those that are also within a minimal clinically important difference (MCID) of the best treatment. Decision rules and ranking systems are compared on stylised examples and 10 NMAs used in NICE (National Institute of Health and Care Excellence) guidelines. Only LaEV reliably delivers valid rankings under uncertainty and has all the desirable properties. In 10 NMAs comparing between 5 and 41 treatments, an EV decision maker would recommend 4–14 treatments, and LaEV 0–3 (median 2) fewer. GRADE rules give rise to anomalies, and, like the probability-based rankings, the number of treatments recommended depends on arbitrary probability cutoffs. Among treatments that are superior to the reference, GRADE privileges the more uncertain ones, and in 3/10 cases, GRADE failed to recommend the treatment with the highest EV and LaEV. A two-stage approach based on MCID ensures that EV- and LaEV-based rules recommend a clinically appropriate number of treatments. For a risk-averse decision maker, LaEV is conservative, simple to implement, and has an independent theoretical foundation.

## Highlights

### What is already known

- A risk-neutral decision-maker should make treatment decisions based on expected value (EV), meaning that the single treatment with the highest expected efficacy from a NMA should be recommended, regardless of uncertainty. In practice, decision-makers may recommend several treatments and take uncertainty into account on an *ad hoc* basis.

### What is new

- We introduce LaEV as a mechanism for risk-averse decision-making, and set out desirable properties of ranking systems. For a ranking to be valid under uncertainty a higher EV must be ranked above a lower one at the same uncertainty, and a lower uncertainty above a higher one at the same EV. We compare LaEV to GRADE and probabilistic rankings. Of the methods examined, only LaEV provides a valid ranking under uncertainty and has all the desirable properties.

### Potential impact for RSM readers

- For a risk-averse decision-maker, LaEV is a reliable, conservative, and easy-to-implement decision metric with an independent theoretical foundation. Adoption of a risk-averse stance might focus attention on more accurate quantification of uncertainty and encourage generation of better quality evidence.

## 1 Introduction

In decision theory a risk-neutral decision-maker bases their recommendations on the ‘expected value’ (EV) of a chosen evaluation function, without consideration of uncertainty. The evaluation function could be:

- A measure of treatment efficacy, for example, probability of an event estimated from a NMA.
- Net Benefit,^1^ which is monetised lifetime health gain minus lifetime costs.
- Or any function of health improvements and adverse events, such as Multi-Criteria Decision Analysis.^2^

The choice of EV as a decision metric is based on a substantial statistical literature^3^ ^–^ ^6^ going back to the 17th century.^7^ In health economic evaluations, EV is regarded as optimal at a societal level^8^ as it delivers a maximally efficient allocation of resources, known as Pareto-optimality.

Faced with multiple options, an EV decision maker should therefore recommend the single treatment with the highest EV, regardless of uncertainty.^9^ In this sense, the EV decision maker is ‘risk-neutral’. In practice, however, decision makers often recommend multiple treatments, and are influenced by the degree of uncertainty in the evidence, suggesting that they are acting as risk-averse decision-makers who have a preference for more certain outcomes. In the UK, for example, multiple treatments have been recommended by NICE in both Multiple Technology Assessments,^10^ ^,^ ^11^ and more often in clinical guidelines.^12^ ^–^ ^14^ This seems to be done on an *ad hoc* basis, usually when treatments have similar efficacy, reflecting a desire to keep clinical options open in case of patient differences in efficacy or side effects, factors that are seldom included in the formal decision model.

Uncertainty has also been treated in an *ad hoc* and even ambiguous manner in NICE’s official documents. The 2022 NICE manual for health technology evaluation (Section 6.3.5) requires that ‘the degree of certainty or uncertainty around the ICER’ (Incremental Cost-Effectiveness Ratio) be taken into account.^15^ The general intention is that less should be paid for an uncertain technology (Section 6.2.34), representing a ‘risk-averse’ approach. However, if it is considered that better evidence is unlikely to be forthcoming, NICE may set a *higher* willingness-to-pay threshold: this is regarded as appropriate in Highly Specialised Technology evaluations for rare diseases (Section 7.1). In this case decision-makers faced with an uncertain intervention are willing to pay above the standard tariff per QALY gained, representing a risk-seeking stance. Thus, while the general decision-making position in NICE guidance is risk-neutral EV, the behaviour of NICE committees and NICE’s own documentation depart from EV in *ad hoc* and seemingly unprincipled ways.

Uncertainty in treatment rankings has also attracted the attention of NMA methodologists.^16^ ^–^ ^19^ Besides ranking by EV, properties of alternative ‘treatment hierarchies’, or treatment rankings have been examined formally,^20^ including: the probability of having the highest value, Pr(Best); the proportion of competitors that a treatment is superior to, also known as SUCRA (surface under the cumulative ranking curve),^21^ or its equivalent, the P-Score.^22^ The probability that the value of the evaluative function exceeds a certain threshold, abbreviated here as Pr(*V* > *T*), has also been studied.^23^ ^,^ ^24^

It has been proposed that these and other^25^ probability-based metrics, which, unlike EV, take uncertainty into account, could help guide NMA treatment decisions.^20^ ^,^ ^26^ ^,^ ^27^ However, by themselves, ranking metrics do not define how many—or even if any—of the top-ranked treatments should be recommended. In an EV context, this can be addressed by a two-stage approach, suggested in earlier work on threshold analysis.^28^ The first stage identifies treatments that are superior to a standard reference treatment; the second selects all those that are also within a MCID margin of the best treatment. The GRADE Working Group adopted a similar multi-stage scheme: in Stage 1, it picks out treatments where Pr(*V* > *T*) exceeds a standard probability criterion such as 0.975.^29^ Subsequent stages identify a subset of these treatments none of which are better than any other on the same criterion.

It has been said that ‘each ranking metric … answer[s] a specific treatment hierarchy question, and … every ranking metric provides a valid treatment hierarchy for the corresponding question’,^20^ a sentiment repeated in subsequent papers.^24^ ^,^ ^27^ ^,^ ^30^ However, any number of rankings and decision schemes could be proposed: we therefore need to ask: what are the properties that would make a ranking ‘valid’ under uncertainty? And what is the ‘treatment hierarchy question’ that decision makers *should* be trying to answer? After all, both Pr(Best) and SUCRA can have the perverse effect of privileging treatments with more uncertain effects.^18^ ^–^ ^20^

In this article, we attempt to identify and evaluate an alternative to EV, which provides a rational approach to multiple treatment recommendations and, at the same time, penalises uncertainty. We will propose a metric based on Bayesian statistical decision theory,^31^ ^,^ ^32^ in which the expected loss arising from taking a decision under uncertainty is subtracted from the EV: We call this the Loss-adjusted EV (LaEV).

We begin by defining three ranking and three decision methodologies and illustrate their properties through stylised examples. We define a key property required for a ranking system to be valid under uncertainty and suggest other desirable properties. The methodologies are then applied to 10 NMAs conducted by NICE guideline developers and published in NICE guidelines and associated publications.

## 2 Methods: Decision rules and ranking systems

In this section, we outline a range of existing decision rules and ranking systems and propose a new metric, LaEV. We begin by defining the standard risk-neutral EV approach and a two-stage extension that allows for multiple recommendations. We then define the GRADE method for a ‘minimally contextualised framework’,^29^ followed by LaEV. Finally, we define three probability-based ranking systems, all familiar from previous literature, and present them as decision rules, in a way that facilitates comparison with the other methods.

### 2.1 The NMA model and its relation to decision outcomes

We assume a standard reference treatment 1, and ‘new’ treatments 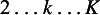 . The NMA estimates a joint probability distribution for 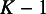 relative treatment effect parameters 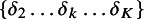 on the linear predictor scale. Given an estimate of the outcome on the reference treatment, 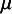 , we obtain estimates of the absolute efficacy of all 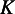 treatments on this scale as 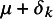 . By definition 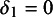 . Via an appropriate link function 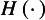 , these parameters inform distributions for the absolute and relative efficacy of treatments on the natural scale. We write 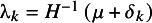 for the absolute efficacy of treatment 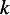 on the natural scale, and 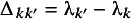 for the relative effect of 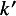 compared to 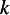 .

For a continuous outcome, the link function 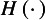 is the identity link, and the absolute and relative effects on the natural scale are the same as those on the linear predictor scale. For a probability outcome, a common choice is the logit link function, and the parameters 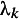 and 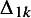 represent probabilities and differences in probabilities respectively. We note that 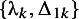 are not point estimates but instead parameters with a joint probability distribution that encapsulates uncertainty, as informed by a Bayesian or frequentist NMA along with a baseline model for the target population.^37^ For avoidance of doubt, it is assumed throughout that all references to variance refer to parameter uncertainty, not heterogeneity. A more general interpretation is suggested in Section 6.

### 2.2 Risk-neutral expected value

#### 2.2.1 Expected value decision rule

The EV decision rule is based on two criteria. We recommend treatments that (i) are at least as ‘good’ as the reference treatment and (ii) are within some threshold 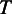 of the best treatment. Comparisons are made based on the EV of the relative treatment effects, 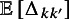 . This is a posterior expectation, conditional on the data. We assume that the outcome of interest is ‘good’, such that 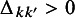 indicates that treatment 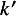 is more effective than treatment 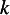 . Therefore, we define the best treatment, 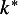 , as the treatment with the highest expected relative effect 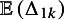 . The EV decision rule can then be expressed as follows:

Recommend any treatment 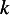 that satisfies both

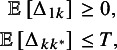

where 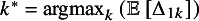 and 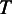 is some appropriate threshold on the natural scale. Since 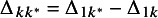 , the risk-neutral EV decision rule can be expressed equivalently as a single criterion,

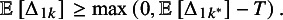



In practice it can be helpful to envisage the rule being applied in two stages. In Stage 1, the evaluative function 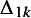 identifies treatments that are non-inferior to reference treatment 1. In Stage 2, the evaluative function 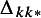 is used to identify treatments within *T* of the best treatment.

#### 2.2.2 Defining a decision threshold

The threshold 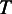 represents the maximum amount by which a treatment can be inferior to the best treatment and still be recommended. As suggested in earlier work on threshold analysis^28^ and by the GRADE Working Group,^29^ a natural choice for 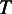 is the MCID, although any measure of clinical importance or non-inferiority could be used as long as it can be expressed on the natural scale.

For probability outcomes, the MCID can be expressed in terms of relative risk. Suppose that 
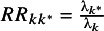
 is the minimal clinically important relative risk chosen by the decision-maker. To find the equivalent threshold 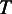 , we set 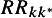 equal to the maximum value at which treatment 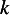 will still be recommended, which by definition must satisfy 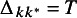 . Substituting 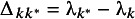 and using 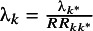, we find
(1)

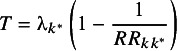



Values of 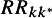 such as 1.25 or 1.5 would be typical.^33^ Note that to evaluate Equation (1) for a given relative risk MCID, we treat 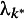 as a constant, for example, by plugging in its EV. Equivalent transformations will be required if the MCID is expressed as an odds ratio, log odds ratio, or probit difference. For example, if the decision-maker chooses a log odds ratio of 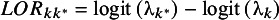 , then the threshold is given by

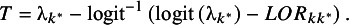



### 2.3 Loss-adjusted expected value

The risk-neutral decision rule in Section 2.2 is based on EV and does not account for the uncertainty in the parameters 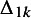 and 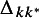 . We propose a risk-averse decision rule that accounts for the expected loss associated with making decisions under uncertainty. The rule is based on two criteria, analogous to those for the risk-neutral EV in Section 2.2. For each criterion, we adjust the EV by the expected loss associated with that decision.

#### 2.3.1 Expected loss of making decisions under uncertainty

The first criterion requires that a recommended treatment should be at least as effective as the reference treatment. Over the distribution of 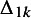 , positive values indicate treatment 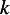 is more effective than the reference so there is no loss in recommending 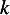 over treatment 1. However, in the region that 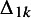 is negative we would obtain a higher payoff (equal to 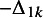 ) if we recommended the current standard treatment. The expected loss from selecting treatment 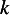 based on the first criterion (i.e., relative to the reference treatment) is therefore
(2)

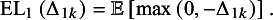



The second criterion ensures that we do not recommend treatments that are worse than the best treatment 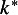 by more than the threshold 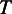 . Over the distribution of 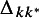 , values less than 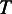 indicate that the efficacy of the best treatment 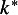 does not exceed 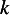 by more than the threshold, so there is no loss associated with recommending treatment 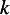 . For values of 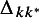 greater than 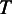 we would obtain a higher payoff (equal to 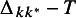 ) if we did not recommend treatment 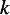 . Therefore, the expected loss associated with this criterion is
(3)

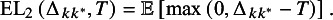



The expected loss arising from making a decision under uncertainty, a decision based on the imperfect evidence currently available, is equivalent to the expected gain if all uncertainty was removed before making the decision. In Bayesian Decision Theory this is known as the expected value of perfect information (EVPI).^31^ However, these concepts usually refer to the value of decisions between multiple treatments, whereas here interest is focussed on pairwise decisions: first on the value of a decision to recommend a single treatment when compared to the reference, and second to recommend a treatment when compared to the ‘best’.

#### 2.3.2 Loss-adjusted expected value decision rule

To construct our LaEV decision rule, we penalise the EV involved in each criterion by its associated expected loss. For the first criterion, larger values of 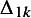 indicate that treatment 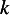 is more effective than the reference. Therefore, to penalise the EV of 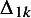 we subtract the expected loss in Equation (2). For a treatment that fulfils the first EV criterion, we can interpret the LaEV as moving the EV towards the null. For the second criterion, the smaller the value of 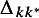 , the closer treatment 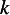 is to the best treatment. That is, smaller values of 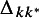 indicate greater efficacy of treatment 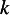 (i.e., closer to the best treatment in efficacy). Therefore, to penalise the EV of 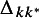 , we add the expected loss associated with this criterion (Equation (3)). As the degree of uncertainty and therefore the expected loss increase, the LaEV increases towards the threshold T. Once LaEV exceeds this threshold, a treatment that would be recommended under EV is no longer recommended under LaEV. Our LaEV decision rule can then be written as follows:

Recommend any treatment 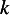 that satisfies both

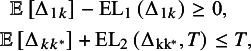

where 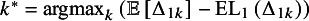 and 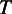 is some appropriate threshold on the natural scale. We refer to the left-hand side of each expression as the LaEV. Once again, the two criteria would be applied in a two-stage process.

For this decision rule, the best treatment 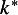 is defined as the one that maximises the LaEV of 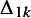 . It is possible that this is different from the treatment with the highest EV used in Section 2.2. However, this is not the case for any of the examples in this article, so for simplicity we use the same notation for both.

Both EV and LaEV depend on the choice of reference treatment. However, both the EV and the LaEV attaching to each treatment are independent of the EV and LaEV attaching to every other treatment. This is an important property, as noted in discussions of value-based pricing.^34^

### 2.4 GRADE Working Group method

In the GRADE multi-stage process for drawing conclusions from an NMA, within what they term a ‘minimally contextualised framework’,^29^ treatments start in Category 0. The process then identifies the set of treatments that are superior to the reference treatment by a threshold margin 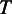 , with probability 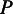 . In other words, all treatments 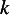 conforming to 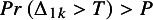 , for example, with 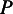 set at the standard benchmark 0.975, and 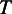 set to the MCID. These treatments are promoted to Category 1. At the second step, any Category 1 treatment 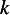 is promoted to Category 2 if it is superior to at least one other Category 1 treatment by the same criteria. The process continues to Category 3 or more, until we are left with a set of treatments none of which are superior to any other by the margin 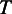 with probability 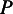 . Finally, the decision rule is to recommend all treatments in the highest category. (In practice, checks for evidence inconsistency and certainty ratings may intervene before recommendations are made). The values of 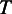 and 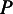 can be changed but are assumed to stay the same within each evaluation.

### 2.5 Probability-based ranking systems

To compare to the decision rules described above, we examine three probability-based ranking approaches: the probability of being best, Pr(Best),^35^ the SUCRA,^21^ and the probability that the value exceeds a threshold, Pr(V > *T*). In the latter case, the decision maker ranks treatments according to the probability that their relative treatment effect exceeds a given threshold, 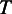 .^24^ The three ranking metrics are defined as follows:

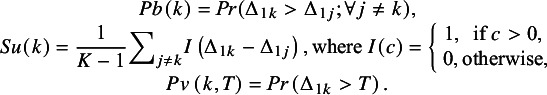



Note, that to implement Pr(V>T) where 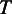 is a relative risk MCID, the RR is relative to reference treatment 1.

The three probability-based ranking systems are not decision rules, but the rankings can be compared to rankings generated by EV, LaEV, and GRADE. To help readers assess how they might perform as decision rules, and to aid comparison with EV-based decisions, we report the 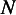 most highly ranked treatments in each NMA, where 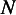 is the number recommended by the EV decision rule.

## 3 Illustration of properties of ranking methods in stylised examples

In the following, we present a set of four hypothetical scenarios to illustrate, compare and contrast the properties of the alternative decision rules and ranking methods. The scenarios are explained alongside the results. WinBUGS code for each illustration is available in the Supplementary Material.

### 3.1 Illustration 1: Impact of uncertainty on EV, LaEV, and Pr(V>T)

**Figure 1:**
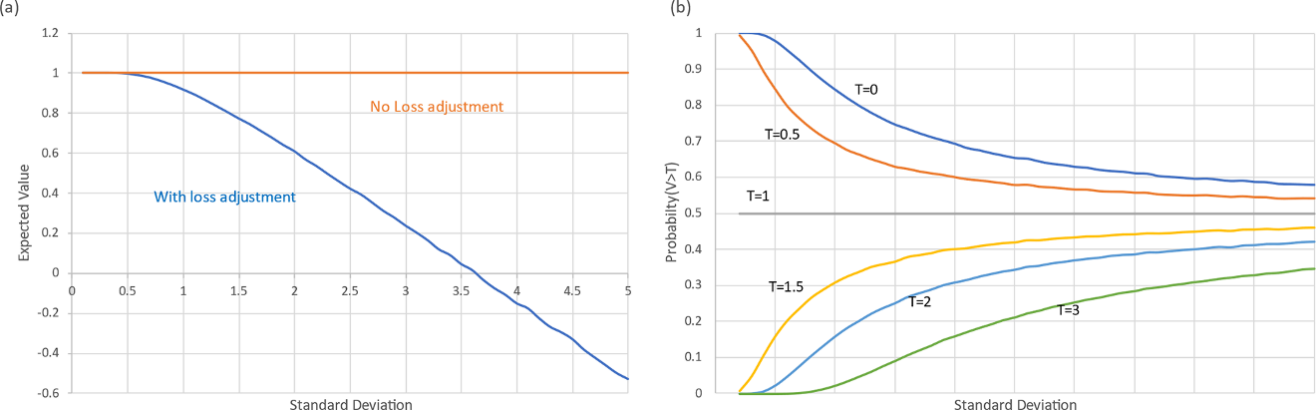
Evaluative function with mean 1.0 and SD varying from 0.1 to 5. (a) Impact of uncertainty on expected value with and without loss-adjustment. (b) Impact of uncertainty on Pr(V > T), the Probability that the value exceeds a threshold, T.

Consider a one-stage two-choice decision involving the relative treatment effect of a single new treatment 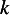 against a standard, and an evaluation function with uncertainty distribution 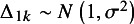 . This represents the uncertainty around the relative effect estimate of a treatment *k* compared to reference treatment 1 on the natural scale. As we vary the 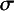 in the data, there is no effect on the EV, but LaEV declines, slowly at first until 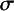 is about 1, at which point it falls off in a roughly linear fashion, reaching half its value at 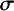 = 2.3, and turning negative at 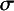 = 3.6. (Figure 1a). At this point the decision-maker would choose the reference treatment.

Pr(*V* > *T*) also declines as 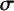 increases, but only when *T* < EV. Otherwise, it rises if *T* > EV, or remains constant at 0.50 if *T* = EV (Figure 1b). Pr(*V* > *T*) therefore does not generate a ranking suitable for routine use. GRADE rules, which take the form: ‘select if Pr(*V* > *T*) > *P*’ are similarly limited.

**Figure 2:**
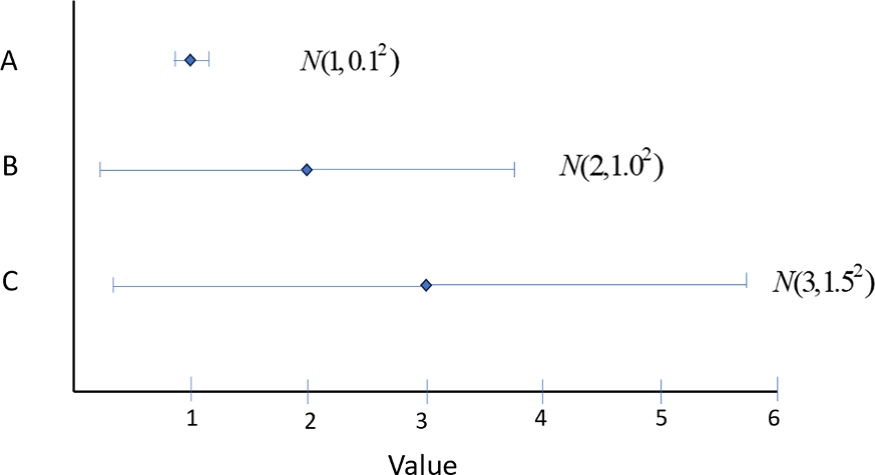
Forest plot showing expected value and 95% credible intervals of three treatments, A, B, C. The probability that the value of A exceeds zero is virtually 1, while the probability that the value of B and C exceed 1 is equal at 0.977. Pr(V > 0) would rank them A, B = C, with metrics (1, 0.977, 0.977). An LaEV decision maker would rank them C, B, A with metrics (2.99, 1.99, 1.0), almost identical to an EV decision maker (3.0, 2.0, 1.0).

### 3.2 Illustration 2: Counter-intuitive properties of Pr(V>T)

Even when EV > *T*, Pr(*V* > *T*) can deliver counter-intuitive rankings. Figure 2 portrays the value distributions of three treatments, A, B, and C, with EVs 1.0, 2.0, 3.0. While A has the lowest EV, the uncertainty in A is negligible, and the probability that *V* > 0 is virtually 1. However, B and C both have an SD that is exactly one half of their EV, so the probability that *V* > 0 is equal at 0.977. Pr(*V* > 0) therefore ranks them (best to worst) A, B = C. In this case, Pr(*V* > *T*) correctly privileges the least uncertain treatment, but it fails to produce a rational decision because it does not reflect the extent of gain or loss, only its probability. In contrast, a LaEV decision-maker, would rank them C, B, A with metrics (2.99, 1.99, 1.0), the same ranking as an EV-based decision, and with almost identical metrics.

### 3.3 Illustration 3: Anomalies in GRADE decision rules

**Figure 3:**
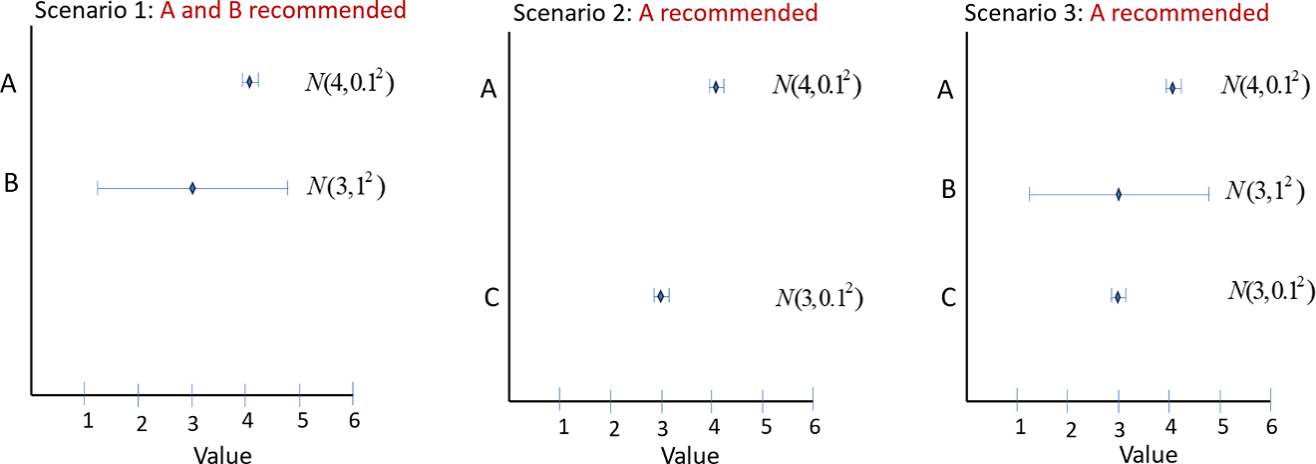
Forest plot showing expected value and 95% credible intervals of three treatments, A, B, C. In Scenario 1, treatments A and B have reached GRADE Category 1 because Pr(V > 1) > 0.975, the MCID being 1. Because A is not superior to B by 1 with Probability 0.975, both A and B remain in Category 1 and are recommended. In Scenario 2, A is superior to C: A is promoted to Category 2 and is recommended, but C is not. In Scenario 3, A is superior to C and is promoted, while B is not. Whether or not B is recommended depends on the presence of C, even though C is never recommended.

Figure 3 portrays three scenarios where GRADE rules are implemented with an MCID = 1 and a probability threshold *P* = 0.975. In Scenario 1 the highly uncertain treatment B is recommended along with A, while in Scenario 2, the much more certain treatment C is *not* recommended, although it has the same EV as B. A treatment that reaches Stage 2 is therefore more likely to be recommended if it is uncertain.

In Scenario 3, all three treatments are compared. In contrast to Scenario 1, where both A and B are recommended, in Scenario 3 only A is recommended, as it is superior to C and is therefore promoted to Category 2. The recommendation of treatment B depends on the presence or absence of treatment C, even though C would not be recommended in any of these scenarios.

**Figure 4:**
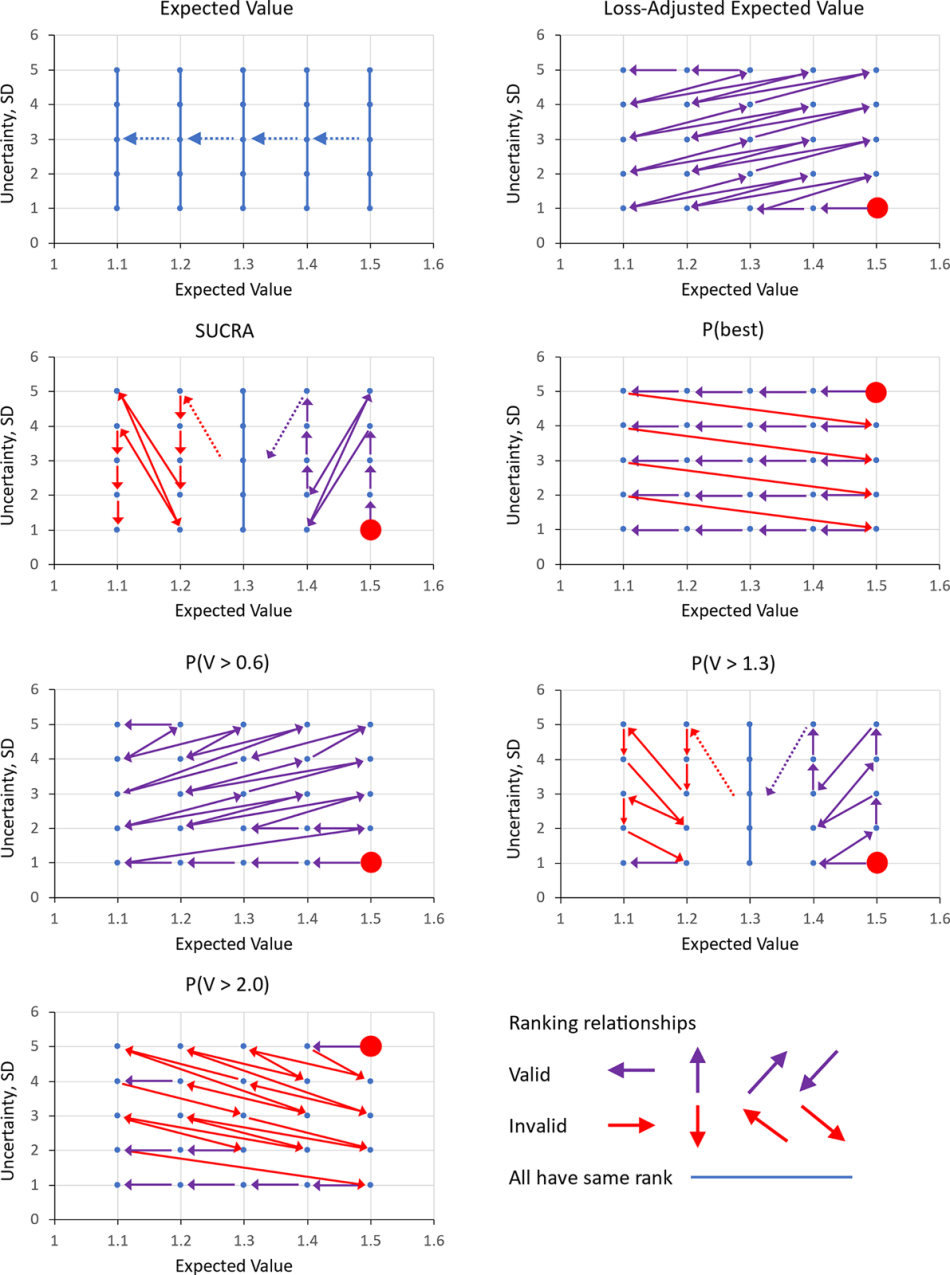
Twenty-five treatments in a 5 × 5 grid with EVs 1.1, 1.2, 1.3, 1.4, 1, 5, and SDs 1, 2, 3, 4, 5. Rankings generated by seven metrics: EV, LaEV, SUCRA, Pr(Best), Pr(V > 0.6), Pr(B > 1.3), Pr(V > 2.3). Arrows start from the highest ranked treatment, marked with a red blob, and point to the 2nd ranked, then the 3rd ranked, and so on. Every treatment must be ranked in order. Treatments linked by a blue line are of equal rank. Valid rankings (coloured purple, see Panel 8) must start at the bottom right and end at the top left. Further, they can only point leftwards, upwards, bottom-left to top-right, or top-right to bottom-left. Arrows pointing downwards (red) are invalid because they imply a higher ranking for a more uncertain treatment with the same EV. Likewise, arrows pointing Rightwards are invalid as they imply a higher ranking for a treatment with a lower EV at the same SD. Arrows running top-left to bottom-right imply higher ranking for treatments with both lower EV and higher SD. Arrows pointing bottom-right to top-left are also invalid because they skip over treatments that either have higher EV with the same uncertainty, or lower SD with the same EV, or both.

### 3.4 Illustration 4: Properties of a valid ranking system in response to uncertainty

Here we consider a (one-stage) ranking of 25 treatments with evaluation functions distributed 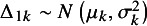 arranged in a five-by-five grid with mean 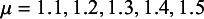 and 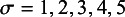 . The rankings of the 25 treatments by EV, LaEV, SUCRA, Pr(Best), Pr(*V* > 0.6), Pr(*V* > 1.3), and Pr(*V* > 2.3) decision rules are presented in a series of grid plots (Figure 4), in which arrows point from highest ranked treatment to the 2nd, then the 3rd, and so on. For a ranking system to be valid under uncertainty, treatments with a higher EV must be ranked above those with a lower EV and the same SD; and those with a lower SD must be ranked above treatments with a higher SD and the same EV. Thus, the arrows must start at the lower right corner and end at the top left.

Based on this simple test, EV, SUCRA, Pr(Best), Pr(*V* > 1.3) and Pr(*V* > 2.0) all generate invalid rankings under uncertainty. Only LaEV and Pr(*V* > 0.6) generate exclusively valid rankings.

## 4 Preferred properties and attributes of treatment rankings

Before turning to real examples, we summarise some preferred properties of decision rules and the treatment rankings under uncertainty, based partly on the illustrative examples. The results are set out in Table 1.

**Table 1:** Performance of alternative ranking methods regarding preferred properties. Properties marked with an asterisk are considered essential.

Property or attribute	EV	GRADE	Pr(Best)	SUCRA	Pr(*V* > *T*)	LaEV	Comments
Valid ranking in the face of uncertainty*	N	N	N	N	N	Y	Illustration 4
Method should generate recommendations, not just rankings*	Y	Y	N	N	N	Y	The probabilistic rankings by themselves do not specify how many, or even if any, treatments should be recommended
Methods that penalise uncertainty should only recommend as many, or fewer treatments, than EV, never more	n/a	N	N	N	N	Y	The probabilistic rankings do not specify upper or lower limits on how many treatments would be recommended
Methods should not depend on arbitrary probability cutoffs	Y	N	N	N	N	Y	Pr(Best), SUCRA, and Pr(*V* > *T*) would require arbitrary probability cutoffs if they were to be used as decision rules
Metric should be in same units as evaluation function	Y	N	N	N	N	Y	This facilitates the use of clinically interpretable benchmarks, such as MCID
Must reflect extent of loss, not just its probability*	N	N	N	N	N	Y	Illustration 2. Also see Ref. 34
Metric for each treatment should be independent of the number of alternative treatments, and the value of metrics for other treatments *	Y	?	N	N	Y	Y	The Pr(*V* > *T*) metric underlying GRADE is independent, but its decision rule makes GRADE decisions dependent on the presence or absence of treatments that would not be recommended (Illustration 3). See Ref. 34 for a similar argument for independence
Methods should have independent theoretical support*	Y	N	N	N	N	Y	Only EV and LaEV have independent theoretical justifications, in the contexts of risk-neutral and -averse decision making, respectively.^34^ ^,^ ^50^

*Note:* GRADE Working Group minimally contextualised framework; Pr(Best), probability best; SUCRA, surface under the cumulative ranking curve; Pr(V > T), probability that evaluative function exceeds threshold T; LaEV, loss-adjusted expected value.

**Table 2:** *NICE guideline smoking cessation.^14^* Outcome is risk of cessation. MCID based on RR = 1.50, or *T* = 0.139 on the probability scale. All the ranks are those generated by an EV ranking. For example, the five treatments ranked highest by GRADE and Pr(*V* > *T*) are the treatments ranked 9, 6, 7, 3, 4 by EV: the five ranked highest by SUCRA are ranked 1, 2, 3, 4, 6 by EV. The EV columns show the posterior means and standard deviations of the evaluative functions in Stages 1 and 2, ( 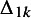 *and* 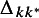 ). Treatments meeting the Stage 1 and Stage 2 decision criteria are shaded. For the ranking systems in Stage 2, we have highlighted the six highest-rank treatments, because six treatments are recommended by EV. Note that in Stage 1 treatment effects are relative to placebo (treatment 1), which is therefore excluded from the ranking; it is however included in Stage 2

Treatment (numbering as in NICE guidelines)	Stage 1 Decision rules							Ranking systems			Stage 2 Decision rules					Final GRADE (0.975) category 1
	EV			LaEV		GRADE (0.975) Category 1					EV			LaEV		
	Rk	EV	sd	Rk	LaEV	Rk	Pr(*V* > *T*)	P(Best)	SUCRA	Pr(*V* > *T*)	Rk	EV	sd	Rk	LaEV	
Buproprion + NRT L&S, 11	1	0.30	0.12	1	0.30	9	1.000	1	1	9	1	0.00	0.00	1	0.00	9
E-cigarette + NRT L/S, 14	2	0.23	0.10	2	0.23	6	1.000	2	2	6	2	0.07	0.15	2	0.10	6
E-cigarette, 9	3	0.21	0.07	3	0.21	7	1.000	4	3	7	3	0.09	0.13	3	0.12	7
Varenicline + Buproprion, 13	4	0.21	0.07	4	0.21	3	0.997	3	4	3	4	0.09	0.14	4	0.13	3
Vareniciline + NRT L/S, 12	5	0.19	0.06	5	0.19	4	0.992	5	6	4	5	0.11	0.13	6	0.15	4
NRT long and short, 6	6	0.19	0.04	6	0.19	5	0.991	6	5	5	6	0.11	0.12	5	0.15	5
Varencline, 8	7	0.15	0.02	7	0.15	1	0.991	12	7	1	7	0.15	0.12	7	0.21	1
Buproprion + NRT L/S, 10	8	0.11	0.03	8	0.11	10	0.987	7	8	10	8	0.19	0.12	8	0.27	10
NRT long/short, 5	9	0.10	0.01	9	0.10	2	0.976	8	9	2	9	0.20	0.12	9	0.29	2
Buprioprion, 7	10	0.09	0.01	10	0.09	8	0.963	11	10	8	10	0.21	0.12	10	0.31	
No drug treatment, 2	11	0.05	0.02	11	0.05	12	0.251	13	11	12	11	0.25	0.12	11	0.38	
Wait list, 3	12	0.03	0.05	12	0.02	11	0.227	9	12	11	12	0.27	0.13	12	0.41	
Placebo, 1	–	–	–	–	0 (R)	–	–	–	–	–	13	0.30	0.12	13	0.46	
Usual care, 4	13	−0.04	0.01	13	−0.07	13	0.000	10	13	13	14	0.34	0.12	14	0.53	

*Abbreviations:* NRT, nicotine replacement therapy; L&S, long and short acting; L/S, long/short acting; Rk, Rank; R, reference treatment.

## 5 Results on NICE guidelines

In this section, we apply the six ranking systems, three with decision rules and three without, to 10 example NMAs from NICE guidelines. We used the original WinBUGS code, data, and initial values from the guidelines (available https://www.bristol.ac.uk/population-health-sciences/centres/beam-centre/mpes/nice/reportsandpublications.html), discarding the same number of burn-in samples. Additional code, shown in the Supplementary Material, generated results for decision rules and rankings. Results were based on 600,000 samples from the Bayesian posterior distribution.

### 5.1 Smoking cessation

**Figure 5:**
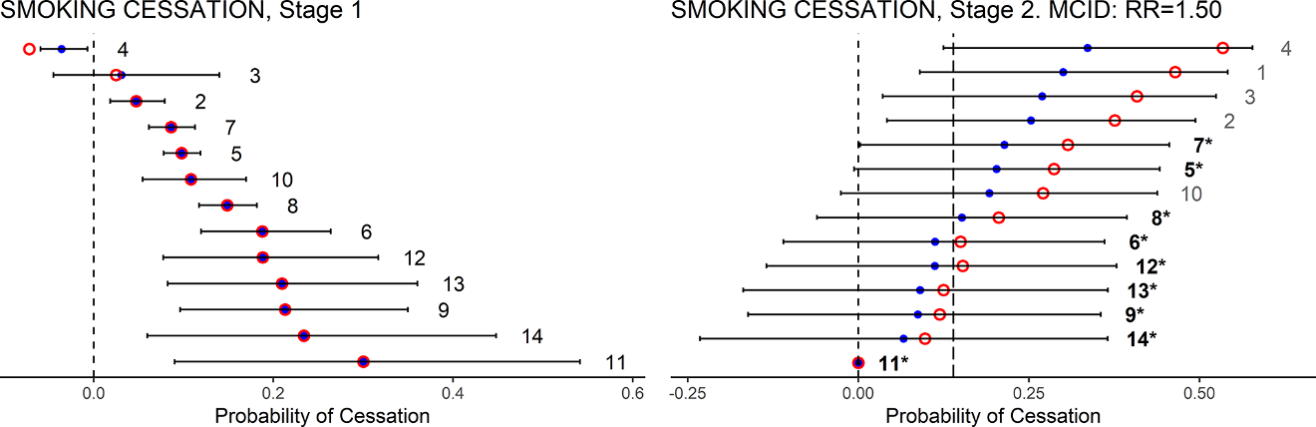
Smoking cessation. Caterpillar plots of the EV (blue dots) and its 95% CrI, and LaEV (red circles) of the Stage 1 and Stage 2 evaluation functions, ( 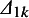 *and* 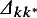 *). Also shown: the coding of treatments in the NICE guidelines; the MCID at Stage 2 (dashed line to the right). Treatments recommended are those with EV, or LaEV, less than the MCID threshold in Stage 2. GRADE recommended treatments are those in bold and marked with asterisks.*

The 2021 NICE Guidelines *Tobacco: prevention of uptake, promoting quitting and treating dependence* ^14^ included an NMA of 13 classes of treatments for smoking cessation against placebo. The trial outcome was the probability of cessation. The results of both Stage 1 and Stage 2 calculations appear in Table 2. Caterpillar plots (Figure 5) show the mean (EV) and uncertainty (95% CrI) in the Stage 1 and Stage 2 evaluation functions ( 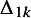 and 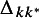 ). Also shown are the LaEV of each treatment. We have applied the EV and LaEV to all treatments at both stages for illustrative purposes: in practice only treatments satisfying the Stage 1 criteria would go on to Stage 2.

In Stage 1, all but one of the 13 active treatments are more effective than placebo based on EV. Note that loss adjustment has virtually no impact on the Stage 1 valuations. This is because, although there is considerable uncertainty in the expected treatment effects, there is very little decision uncertainty: the EVs are so far from zero that the expected loss attaching to choosing each treatment over the reference treatment is negligible. Accordingly, LaEV picks out the same treatments as EV (see Table 2 and Figure 3). In Stage 2, based on EV, the best treatment is joined by five other treatments that are not worse than the best treatment by more than the MCID (RR = 1.50), while LaEV picks out four of these.

Application of the GRADE decision rules with the same MCID and a 0.975 cut-off results in 9 treatments reaching Category 1. GRADE attributes the highest ranks to treatments ranked 9, 6, 7, 3, 4 by EV, because these have exceptionally low SD. Note that if a *P* = 0.50, ‘balance of evidence’ probability had been employed, instead of 0.975, then the effect of GRADE would be identical to EV. This is what would be expected unless the distributions of the evaluative functions are highly asymmetrical. As none of the nine Category 1 treatments are significantly better than any others by an RR of 1.50, none were promoted to Category 2, and all would therefore be recommended. However, while the ranking by GRADE is quite different to the ranking by EV, the nine treatments recommended by GRADE are among the 10 most highly ranked on EV.

SUCRA delivers a ranking that is very close to EV, while the Pr(Best) ranking departs from EV quite markedly. However, if SUCRA and Pr(Best) decision makers were to recommend the same number of treatments as an EV decision maker, they would choose the same six treatments. A Pr(*V* > *T*) decision maker would recommend only four of the treatments recommended by EV.

### 5.2 Other NICE guidelines

Detailed results, references, and commentary for a further 9 NMAs from NICE guidelines are given in the Supplementary Material, and all 10 are summarised in Table 3. The 10 NMAs compared between 4 and 40 treatments to the reference treatment. Some of the NMAs incorporate class models, and in some the guideline developers decided between classes of treatments. To improve network connectivity, NMA datasets sometimes include treatments that are excluded from the decision set. In these cases, we have applied rankings and decision rules only to the decision set.

**Table 3:** Summary results on 10 NMAs from NICE guidelines. Treatment recommendations from decision rules (EV, LaEV) at Stages 1 and 2; GRADE Category 1 and final category treatments; and results from ranking systems, Pr(Best), SUCRA, Pr(*V* > *T*). The numbers listed are the treatment rankings under EV. For ranking systems, the *N* highest ranked treatments are listed, where *N* is the number recommended by EV. The summary statistics for GRADE assume a 0.975 probability cutoff throughout

Guideline	MCID	Max	Treatments recommended (number recommended)				Ranking systems: *N* top-ranked treatments		
			(*N*) EV	LaE*V*	GRADE	(*P*)	Pr(Best)	SUCRA	Pr(*V* > *T*)
Smoking cessation	RR 1.5	14	(6) 1–6	(4) 1–4	(9) 1–7, 9, 10	(0.975)	1–6	1–6	3–7, 9
Moderate to severe acne	CfB 25%	27	(14) 1–14	(11) 1–11	(5) 1, 6–9	(0.975)	1–7, 10, 11, 14, 15, 18, 23, 26	1–14	1–14
Mild to moderate acne	CfB 25%	41	(3) 1–3	(2) 1–2	(1) 2	(0.975)	1–3	1–3	2–4
More severe depression	SMD 0.5	27	(5) 1–5	(3) 1,3,2	(6) 1, 3, 6, 7, 9, 10	(0.85)	1–5	1–5	1, 3, 6, 9, 10
Joint replacement	RR 1.5	5	(2) 1–2	(2) 1–2	(1) 2	(0.975)	1–2	1–2	1–2
Headache	Days 0.5	7	(3) 1–3	(3) 1–3	(1) 3	(0.975)	1, 2, 5	1–3	1–3
Social anxiety (treatment)	SMD 0.5	41	(7) 1–7	(5) 1–5	(24) 1–16, 19–22, 24, 25, 27, 29	(0.975)	1–4, 6, 8, 28	1–7	1, 2, 5, 7, 11, 19, 22
Social anxiety (class)	SMD 0.5	17	(9) 1–9	(6) 1–6	(4) 1, 2, 5, 6	(0.975)	1–5, 7, 8, 10, 13	1–9	1–9
Urinary incontinence	RR 1.25	14	(5) 1–5	(2) 1–2	(8) 1, 3, 6–8, 10–11, 13	(0.975)	1–4, 9	1–5	6–8, 11, 13
Tocolytics	Weeks 1	7	(3) 1–3	(3) 1–3	(1) 1	(0.975)	1–3	1–3	1–3
Summary statistics									
Number recommended									
Mean		20.1	5.7	4.1	5.4				
Range (median)		5–41 (15.5)	2–14 (5)	2–11 (3)	0–24 (2.5)				
% of max: Range (median)			7–53 (39)	5–43 (32)	0–64 (19)				

*Abbreviations:* MCID, minimal clinically important difference; Max, maximum number of treatments that could be recommended; *N*, number of treatments recommended by EV; *P*, GRADE probability cutoff; RR, relative risk; CfB, change from baseline; SMD, standardised mean difference.

EV decision-makers would recommend between 2 and 14 interventions (median 5), while LaEV would recommend between 3 and 11 (median 3), between zero and 3 (median 2) fewer than EV. GRADE rules with a 0.975 probability cut-off recommend between zero and 24 treatments (median 2.5), between 9 fewer and 17 more than EV. In 3/10 cases the treatment which was ranked best by EV (and LaEV) was not among the treatments recommended by GRADE. At Stage 1, GRADE privileges more certain treatments at the expense of better EV, as seen in illustration 2. However, at Stage 2, the more *un*certain treatments are recommended as they are less likely to be ‘significantly’ different from the best treatment.

The rankings produced by Pr(Best) and Pr(*V* > *T*) tend to differ from the EV and LaEV rankings, while SUCRA rankings are closer to EV. If we look at the *N* top-ranked treatments, where *N* is the number recommended by EV, SUCRA decision makers would recommend the same treatments in all 10 cases, Pr(Best) decision makers in 6, and Pr(*V* > *T*) decision makers 5.

## 6 Discussion

This article attempts to define a rational basis for recommending more than one treatment on the basis of NMA evidence while penalising uncertainty. This represents a risk-averse decision-making position, in contrast to the standard EV approach. Using stylised illustrations and real examples from NICE Guidelines, we have compared EV, LaEV, and GRADE decision rules. The performance of ranking systems based on Pr(Best), SUCRA, and Pr(*V* > *T*) has also been documented in view of the growing literature proposing that probabilistic rankings can help inform recommendations.^20^ ^,^ ^23^ ^–^ ^27^ ^,^ ^36^

For a ranking to be valid under uncertainty a treatment with a higher EV must always be ranked above a treatment with a lower EV and the same uncertainty, and a treatment with less uncertainty must always be ranked above a treatment with more uncertainty at the same EV. Of the methods examined only LaEV provides a valid ranking by this criterion. Pr(V > *T*) is valid only if EV > *T for all treatments*, a property which blocks its use in routine applications, and which is inherited by the GRADE Working Group rules for a minimally contextualised framework. Although SUCRA usually generates a ranking close to EV in real examples, except when treatments differ substantially in uncertainty, it cannot be relied on to produce valid rankings under uncertainty, and it possesses none of the preferred properties. The probabilistic ranking systems and GRADE all take uncertainty into account, sometimes in irrational ways, but they do not always penalise it. They may register the probability of loss, but not its extent. Their fundamental drawback is that they do not distinguish between uncertainty in treatment effects from and uncertainty in decision. Possibly, SUCRAs could be modified or weighted to improve their response to uncertainty, but it is not clear that this would change their rating on the Table 1 criteria.

Because the EV and LaEV decision metrics are on the same scale as the evaluative function, they can access the MCID. This provides a natural basis for deciding how many treatments besides the best treatment should be recommended. MCID has been used in this way in NMA threshold analyses^28^ and has had a similar role in Bayesian sensitivity analyses more generally.^37^ Because expected loss is always positive (for treatments better than the reference), LaEV decision rules cannot recommend more treatments than EV, and any approach that penalises uncertainty should have this property. The number of treatments recommended by GRADE sometimes exceeds EV, and is effectively arbitrary, subject only to the choice of probability cutoff. SUCRA delivers rankings close to the EV ranking in real examples, Pr(Best) and Pr(*V* > *T*) less so, but arbitrary cutoffs would again be required to control the number of treatments recommended by all three probabilistic ranking methods.

A further property of the GRADE rules is that it is not possible to recommend the standard reference treatment alongside any other treatments. This is because treatments that are better than treatment 1 by the stated criterion are promoted to Category 1, leaving treatment 1 behind in Category 0. It therefore seems that reference treatment 1 can therefore only be recommended by default if no other treatment betters it (on the stated criterion).

Adoption of any risk-averse decision rule would put a new spotlight on uncertainty and its sources. Much of the uncertainty in model parameters originates in sampling error in their estimation, but variation arising from random effects models also contributes, representing, perhaps, the uncertain relevance^38^ of evidence from trials with widely dispersed treatment effects. These sources of uncertainty are ‘within’ the decision model and can therefore engage risk-averse methods for decision-making. On the other hand, the use of GRADE certainty ratings^39^ and Risk of Bias tools^40^ identifies further sources of uncertainty which tend to be treated as external or contextual factors that are ‘taken into account’ alongside the results of formal modelling. Model structure and choice of data sources represent further sources of uncertainty outside the decision model, often addressed by sensitivity analyses. Adopting decision rules that penalise uncertainty would encourage investigators to bring all such sources of uncertainty *into* the decision model and would place a premium on statistical methods that reduce between-study heterogeneity, including: informative priors on variance parameters^41^; bias modelling^42^; and methods that increase precision such as multi-level network meta-regression.^43^ Bias models are already in common use in NICE guidelines.

Variation due to between-study heterogeneity has been a major feature in the evidence synthesis and NMA landscape and it is useful to clarify how this relates to the 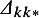 , in which the only variation is parameter uncertainty (Section 2.1). In the case of synthesis of aggregated heterogeneous trials using a random effects model, we assume that the decision maker considers the relation between the target population for decision and the trial populations in the synthesised studies. Based on this, the most relevant output from the meta-analysis is selected to serve as a basis for the decision, such as the random effects mean, the predictive distribution of effects in a new study, or the shrunken estimate from the most relevant study or studies.^44^ ^–^ ^46^ This summary effect measure, 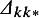 , is therefore a single parameter that already captures heterogeneity appropriately for the decision-maker, and the variance in 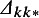 is interpreted as purely parameter uncertainty.

In light of the increasing interest in the precise definition of estimands,^47^ we should also consider how between-individual variation ties in. Assume that an individual patient data NMA results in a model on a linear predictor scale including coefficients for a treatment effect and for both prognostic and effect-modifying variables. Where there is between-individual variation, 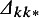 is a population-average marginal treatment effect on the natural scale (i.e., a marginal risk difference for a binary outcome).^48^ This is derived by an integration over the joint covariate distribution, which is typically known, but could include uncertainty. The expectation of this 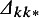 includes more parameters, and has different uncertainty characteristics,^43^ ^,^ ^49^ but remains an expectation over uncertain parameters.

The circumstances under which a risk-averse posture is appropriate remain a matter of debate and beyond the scope of this article. Briefly, an EV (risk-neutral) position is considered appropriate for a decision maker making large numbers of decisions under uncertainty,^50^ for example, a national reimbursement agency. Put simply, the risks ‘average out’. However, for individual patients making a one-time decision, a risk-averse stance—penalising uncertainty—would be justified. Risk aversion is also appropriate for institutional decision-makers if costs or benefits are born by individuals and cannot be transferred,^50^ or where payers have limited budgets.^34^ ^,^ ^50^ Although clinical guidelines may apply to large numbers of patients, guideline development groups typically take one-time decisions. There is empirical evidence that both patients^51^ ^–^ ^53^ and clinicians^54^ are risk-averse when facing health care decisions. How and whether this is relevant to the present article is, however, debatable. Our objective here is to develop a basis for rational decision-making under uncertainty, for application in a policy context. Its relevance to the individual decision-maker, or to theories of human choice behaviour, is beyond the scope of the article.

A limitation of this article is that we have not discussed other approaches to risk aversion in the literature, including: mean-variance trade-offs, methods setting a maximum probability of a poor outcome, and methods where risk aversion is a parameter input. These alternatives have seen limited uptake^34^ and none have been considered in the NMA literature. In most cases, fair comparisons would be difficult to contrive, as additional parameters are required whose values are to some extent arbitrary. A possibly more serious shortcoming is our focus on risk aversion, excluding the potential role of a risk-seeking stance. Prospect Theory asserts that risk posture depends on baseline risk,^55^ and there is evidence that, in health care decisions, individuals are risk-seeking at low levels of baseline health.^52^ ^,^ ^56^ ^–^ ^59^ In the context of Net Benefit analysis this has been addressed by Generalised Risk-Adjusted Cost-Effectiveness (GRACE), in which willingness-to-pay varies with baseline risk.^60^ ^,^ ^61^ It may be, therefore, that LaEV as elaborated here is not suited to life-threatening conditions or where the baseline life expectancy or quality-adjusted life expectancy is low. Whether our proposals can be extended to allow risk posture to depend on baseline health status and, more generally, to evaluations based on Net Benefit, are topics of on-going research.

LaEV appears to constitute a relatively conservative methodology for risk-averse decision-makers. In the 10 examples, it recommended only 0–3 fewer (median 2) treatments than EV. It requires an SD of 2.3 units to halve a single unit of EV and an SD of 3.6 units to entirely neutralise it (Illustration 1). We can therefore anticipate that if LaEV was to replace EV-based decision-making, the impact would be no more than moderate. A more substantial impact would be expected where highly uncertain evidence is used, for example, evaluations based on non-randomised evidence, or ‘unanchored’ comparisons.^62^ This underscores the importance of properly representing uncertainty within the decision model: if this were implemented, routine use of risk-averse decision-making methods might incentivise the production of better quality data,^63^ reversing the trend towards accepting evidence from non-randomised and one-arm studies.^64^

Methods used by guideline developers need to be acceptable to key stakeholders, including professional colleges, manufacturers, health care workers, and patients. Stakeholders require a degree of certainty regarding which methods for health technology assessment are acceptable and how they are to be applied. To achieve this, methods have to meet criteria for transparency and consistency across conditions.^65^ This weighs against methods where parameters can be set in arbitrary ways, therefore against GRADE and against decision rules based on SUCRA or Pr(*V* > *T*) rankings, if they were to be proposed. Also problematic are ranking methods that combine efficacy with other outcomes such as adverse effects, costs, or GRADE certainty ratings, using arbitrary, condition-dependent weightings,^23^ even if they were able to reliably produce valid rankings under uncertainty. More fundamentally, GRADE and the probabilistic ranking systems, and indeed other novel ranking approaches,^25^ ^,^ ^36^ stand outside the standard theory and practice of health evaluation. Indeed, no theoretical basis has been proposed in which any of these methods would represent an optimal basis for decision-making.

In 2001, the Institute of Medicine identified ‘patient centred medicine’ as an objective for improved health in the 21st century,^66^ and this was widely endorsed by research funders and organisations delivering health care. Patient-centric decision-making was seen as an essential component. Given that individuals are generally risk-averse when facing health care decisions, a risk-averse methodology by guideline developers would be a step towards patient-centred medicine. For this purpose, the two-stage LaEV method can be recommended as reliable, conservative, theoretically well-motivated, and simple to implement.

## Supporting information

Ades et al. supplementary material Ades et al. supplementary material

## Data Availability

No new data were created or analysed in this study. The original WinBUGs code, data, and initial values, along with the additional code for rankings and decision, are available at https://url.uk.m.mimecastprotect.com/s/QS4xCXoxxFX1A68YI6fmUWKXoQ?domain=bristol.ac.uk.

## Supplementary material

To view supplementary material for this article, please visit http://doi.org/10.1017/rsm.2025.17.
