# Supplementary material for: Treatment recommendations based on network meta-analysis: Rules for risk-averse decision-makers
Source: Res Synth Methods. 2025 Apr 24;16(3):550–68. doi: 10.1017/rsm.2025.17 (PMC12527546; doi:10.1017/rsm.2025.17)
Supplement: Ades et al. supplementary material [file S1759287925000171sup001.docx]

**SUPPLEMENTARY MATERIALS.**

**Treatment recommendations based on Network Meta-Analysis: rules for risk-averse decision-makers**

**Authors:** A E Ades^1^, Annabel L Davies^1^, David M Phillippo^1^, Hugo Pedder^1^, H Thom^1^ , Beatrice Downing^1^, Deborah M Caldwell^1^, Nicky J Welton^1^.

^1^ Population Health Sciences, Bristol University Medical School, Bristol, United Kingdom

1. **Detailed results on NMAs from NICE guidelines**

**A1: Moderate to Severe Acne**

**A2: Mild to Moderate Acne**

**A3: More Severe Depression**

**A4: Tranexamic Acid in Joint Replacement**

**A5: Headache prophylaxis**

**A6: Social Anxiety (Treatment)**

**A7: Social Anxiety (Class)**

**A8: Urinary Incontinence**

**A9: Tocolytic Therapy**

1. **WinBUGS code for illustrative examples**
2. **WinBUGS code for NICE Guidelines**
3. **References**

**Note on the Supplementary Tables and Figures.**

The ***tables*** show the results of applying the decision rules and ranking systems in a series of NMAs from NICE Guidelines. The Tables show the posterior mean Expected Value (EV) and standard deviation of the evaluative functions at Stages 1 and 2 ($\Delta_{1k}$ and $\Delta_{kk^{*}}$), and the Loss-adjusted EV (LaEV).

All the rankings refer to the ranks generated by the EV (see the caption to Table 2 in the main text).

For GRADE we show which treatments are promoted to Category 1, again referring to treatments by their EV ranking. Also shown is the probability that the evaluative function exceeds the threshold. The final GRADE categorization is shown in the last column on the right. (In every case no treatments were promoted to Category 2, so the Category 1 treatments are those recommended under GRADE.

The rankings of the treatments that would be recommended under the EV, LaEV and GRADE decision rules are shaded. For the three probabilistic ranking systems, we have shaded the N top-ranked treatments, where N is the number of treatments recommended by EV.

Note that in Stage 1 treatment effects are relative to reference Treatment 1, so Treatment 1 is not included in the rankings. It is, however, included in Stage 2.

The ***figures*** are caterpillar plots of the EV (blue dots) and its 95%CrI, and LaEV (red circles) of the Stage 1 and Stage 2 evaluation functions ($\Delta_{1k}$ and $\Delta_{kk^{*}}$), ordered by EV. Also shown: the coding of treatments in NICE guidelines, and the MCID at Stage 2 (dashed line on the right). Treatments recommended are those where the mean of the Stage 2 evaluative function ($\Delta_{kk^{*}}$), or the Loss-adjusted EV, is less than the threshold T, set at the MCID.

LaEV SUPP v8

October 2024

**APPENDIX A: DETAILED RESULTS ON NMAS FROM NICE GUIDEINES**

**A1. Moderate-to-Severe Acne**^1^

**Table S1**

| **Treatment**  (numbering as in NICE guidelines) | **STAGE 1**  **Decision Rules** | | | | | | | **Ranking systems** | | | **STAGE 2**  **Decision Rules** | | | | | **FINAL GRADE**  (0.975)  Category 1 |
| --- | --- | --- | --- | --- | --- | --- | --- | --- | --- | --- | --- | --- | --- | --- | --- | --- |
|  | **EV** | | | **LaEV** | | **GRADE**  (0.975)  Category 1 | |  |  |  | **EV** | | | **LaEV** | |  |
|  | **Rk** | **EV** | **Sd** | **Rk** | **LaEV** | **Rk** | **Pr(V>T)** | **P(Best)** | **SUCRA** | **Pr(V>T)** | **Rk** | **EV** | **sd** | **Rk** | **LaEV** |  |
| Retinoid tcd ≥ 120mg/kg (sc)[o] 9 | 1 | 58.0 | 10.7 | 1 | 58.0 | 1 | 0.999 | 2 | 1 | 1 | 1 | 0.0 | 0.0 | 1 | 0.0 | 1 |
| *PhTh therapy 17 | 2 | 57.6 | 17.4 | 2 | 57.6 | 8 | 0.994 | 1 | 2 | 8 | 2 | 0.2 | 20.1 | 2 | 1.3 | 8 |
| *Nicotinamide[t] 7 | 3 | 49.9 | 13.8 | 3 | 49.9 | 7 | 0.993 | 3 | 3 | 7 | 3 | 8.3 | 17.0 | 3 | 9.7 | 7 |
| *PhTh+photodynamic therapy 16 | 4 | 47.9 | 15.4 | 4 | 47.9 | 9 | 0.984 | 4 | 5 | 9 | 4 | 10.0 | 18.2 | 4 | 12.1 | 9 |
| Retinoid tcd<120mg/kg (sc)[o] 8 | 5 | 47.6 | 14.1 | 5 | 47.6 | 6 | 0.981 | 5 | 4 | 6 | 5 | 10.4 | 17.2 | 5 | 12.3 | 6 |
| *Tetracycline [oral]+PhDy therapy 27 | 6 | 44.8 | 9.5 | 6 | 44.8 | 2 | 0.968 | 6 | 7 | 2 | 6 | 13.2 | 13.1 | 6 | 14.6 |  |
| Lincosamide[t]+Retinoid[t] 22 | 7 | 44.5 | 7.8 | 7 | 44.5 | 3 | 0.965 | 11 | 6 | 3 | 7 | 13.6 | 12.5 | 7 | 14.8 |  |
| BP[t]+Retinoid[t]+Tetracycline[o] 26 | 8 | 43.5 | 7.2 | 8 | 43.5 | 12 | 0.958 | 7 | 8 | 12 | 8 | 14.6 | 8.1 | 8 | 15.0 |  |
| Photodynamic therapy 15 | 9 | 40.5 | 7.1 | 9 | 40.5 | 5 | 0.945 | 18 | 9 | 5 | 9 | 17.6 | 12.1 | 9 | 19.6 |  |
| *No treatment 2 | 10 | 39.4 | 18.7 | 10 | 39.2 | 4 | 0.931 | 14 | 10 | 4 | 10 | 18.4 | 21.2 | 10 | 24.0 |  |
| Azelaic acid[t]+Tetracycline[o] 24 | 11 | 38.4 | 15.8 | 11 | 38.4 | 13 | 0.919 | 10 | 11 | 13 | 11 | 19.6 | 18.8 | 11 | 24.7 |  |
| Retinoid[t]+Tetracycline[o] 25 | 12 | 35.2 | 5.9 | 12 | 35.2 | 11 | 0.802 | 15 | 12 | 11 | 12 | 22.9 | 11.0 | 12 | 26.2 |  |
| Lincosamide[t] 4 | 13 | 34.1 | 6.6 | 13 | 34.1 | 14 | 0.791 | 26 | 14 | 14 | 13 | 23.9 | 11.8 | 13 | 28.1 |  |
| BP[t]+Retinoid[t] 21 | 14 | 34.0 | 11.1 | 14 | 34.0 | 10 | 0.782 | 23 | 13 | 10 | 14 | 24.1 | 15.4 | 14 | 29.8 |  |
| PhCh therapy[red] 12 | 15 | 29.7 | 11.5 | 15 | 29.7 | 16 | 0.675 | 8 | 15 | 16 | 15 | 28.3 | 15.2 | 15 | 36.0 |  |
| Benzoyl peroxide[t] 3 | 16 | 28.9 | 8.6 | 16 | 28.9 | 15 | 0.662 | 9 | 16 | 15 | 16 | 29.4 | 13.7 | 16 | 37.3 |  |
| PhCh+PhTh therapy 14 | 17 | 28.3 | 15.7 | 17 | 28.0 | 17 | 0.587 | 16 | 17 | 17 | 17 | 29.5 | 18.6 | 17 | 39.4 |  |
| *Co-cyprindiol[o] 11 | 18 | 25.0 | 15.5 | 18 | 24.7 | 18 | 0.501 | 21 | 18 | 18 | 18 | 33.0 | 18.8 | 19 | 43.8 |  |
| Tetracycline[o] 10 | 19 | 24.2 | 4.1 | 19 | 24.2 | 19 | 0.423 | 12 | 19 | 19 | 19 | 33.9 | 10.0 | 18 | 45.1 |  |
| BP[t]+Lincosamide[t]+Retinoid[t] 23 | 20 | 23.1 | 7.5 | 20 | 23.1 | 23 | 0.406 | 20 | 20 | 23 | 20 | 35.0 | 13.1 | 20 | 46.7 |  |
| BP[t]+Lincosamide[t] 19 | 21 | 22.7 | 8.4 | 21 | 22.7 | 20 | 0.394 | 17 | 23 | 20 | 21 | 35.5 | 13.6 | 21 | 47.7 |  |
| BP[t]+Macrolide[t] 20 | 22 | 22.1 | 4.8 | 22 | 22.1 | 21 | 0.391 | 13 | 21 | 21 | 22 | 36.0 | 11.7 | 22 | 48.0 |  |
| *BP[t]+Anti-fungal[t] 18 | 23 | 22.1 | 12.2 | 23 | 21.9 | 22 | 0.272 | 24 | 22 | 22 | 23 | 36.2 | 16.2 | 23 | 49.7 |  |
| Retinoid[t] 5 | 24 | 13.1 | 2.5 | 24 | 13.1 | 26 | 0.258 | 25 | 26 | 26 | 24 | 45.0 | 11.0 | 24 | 65.1 |  |
| Macrolide[t] 6 | 25 | 10.9 | 7.3 | 25 | 10.7 | 25 | 0.028 | 19 | 24 | 25 | 25 | 47.2 | 13.0 | 25 | 69.6 |  |
| *PhCh therapy[blue and red] 13 | 26 | 8.8 | 24.8 | 26 | 2.8 | 24 | 0.000 | 22 | 25 | 24 | 26 | 48.8 | 26.5 | 26 | 74.9 |  |
| Placebo (Ref) 1 | - | 0(R) | - | - | 0(R) | - | - | - | - | - | 27 | 58.1 | 10.7 | 27 | 91.2 |  |

Abbreviations: BP Benzoyl peroxide; tcd total cumulative dose; [t] topical; [o] oral; [ph] physical; (sc) single course; ToAc Topical Acid; PhCh Photochemical therapy; PhTh Photothermal therapy; PhDy Photodynamic therapy; Comb. Combined; Rk rank (values are ranks on EV); R Reference Treatment 1.

***Moderate-to-Severe Acne***

***Type of model****:* Random study effects, fixed class effects model with 52 treatments in 26 classes relative to placebo.

***Outcome*:** % Change from Baseline.

***MCID***: 25%, ***GRADE probability cutoff****:* 0.975

***EV and LaEV Decision rules***

*Stage 1.* All 26 treatment classes have both positive EV and positive LaEV

*Stage 2*. EV: 14 treatments including the best treatment are recommended, because they are not worse than the best treatment by more than the MCID. *LaEV:* 11 treatments, including the best treatment would be recommended.

***GRADE***

*GRADE:* 5 treatments qualify and are promoted to Category 1. None of them are significantly better than any other by the MCID. Therefore, none are promoted to Category 2, and all are recommended.

***RANKING SYSTEMS***: Pr(V>T) places treatments ranked 8,7,9,6 on EV among the top 5: these have exceptionally low SD. But both Pr(V>T) and SUCRA pick out the same top 14 treatments as EV. The Pr(Best) ranking is erratic, privileging more uncertain treatments

***COMMENTS:*** During consideration of the NMA results by NICE Guidelines developers, a number of treatments were ruled out on the basis of potential bias reflected in their small sample size. These are marked with an asterisk in Table S1. Note that none of those with small sample size would have been ruled out by LaEV.

**Figure S1.** Moderate to Severe Acne.


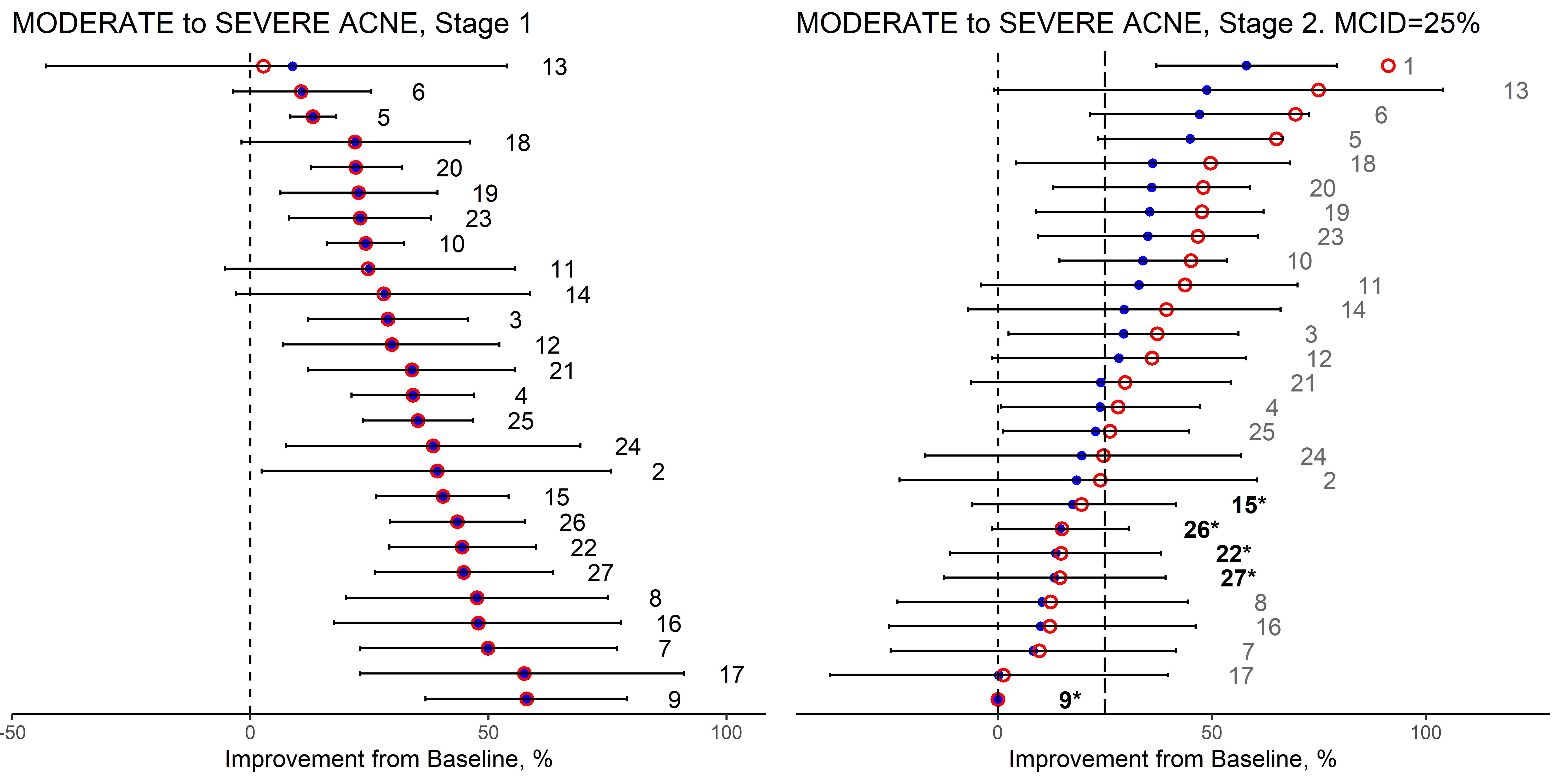


**A2. Mild to Moderate Acne**

**Table S2**

| **Treatment**  (numbering as in NICE guidelines) | **STAGE 1**  **Decision Rules** | | | | | | | **Ranking systems** | | | **STAGE 2**  **Decision Rules** | | | | | **FINAL**  **GRADE**  (0.975)  Category 1 |
| --- | --- | --- | --- | --- | --- | --- | --- | --- | --- | --- | --- | --- | --- | --- | --- | --- |
|  | **EV** | | | **LaEV** | | **GRADE**  (0.975)  Category 1 | |  |  |  | **EV** | | | **LaEV** | |  |
|  | **Rk** | **EV** | **Sd** | **Rk** | **LaEV** | **Rk** | **Pr(V>T)** | **P(Best)** | **SUCRA** | **Pr(V>T)** | **Rk** | **EV** | **sd** | **Rk** | **LaEV** |  |
| *PhCh [red] 23 | 1 | 80.9 | 40.7 | 1 | 80.4 | 2 | 0.984 | 1 | 2 | 2 | 1(R) | 0.0 | 0.0 | 1(R) | 0.0 | 2 |
| *ACNICARE[ph] 15 | 2 | 79.2 | 26.3 | 2 | 79.2 | 3 | 0.938 | 2 | 3 | 3 | 2 | 1.7 | 48.2 | 2 | 11.3 |  |
| *PhTh+photodynamic 26 | 3 | 64.8 | 26.0 | 3 | 64.8 | 4 | 0.934 | 3 | 1 | 4 | 3 | 16.1 | 40.2 | 3 | 27.9 |  |
| *Smoothbeam+PhCh [blue] 27 | 4 | 51.8 | 17.8 | 4 | 51.8 | 1 | 0.916 | 4 | 4 | 1 | 4 | 29.1 | 44.0 | 4 | 48.7 |  |
| Chemical peel[ph] 13 | 5 | 36.9 | 14.1 | 5 | 36.9 | 5 | 0.803 | 5 | 5 | 5 | 5 | 44.0 | 42.7 | 5 | 72.2 |  |
| PhCh[b&r] 21 | 6 | 32.6 | 9.4 | 6 | 32.6 | 6 | 0.792 | 8 | 6 | 6 | 6 | 48.3 | 41.5 | 6 | 79.1 |  |
| *Photodynamic 25 | 7 | 31.0 | 21.8 | 7 | 30.1 | 9 | 0.667 | 11 | 9 | 9 | 7 | 49.9 | 37.3 | 8 | 80.1 |  |
| *No treatment 2 | 8 | 29.6 | 33.2 | 9 | 29.5 | 10 | 0.662 | 10 | 10 | 10 | 8 | 51.3 | 27.5 | 7 | 80.5 |  |
| *BP[t]+Lincosamide[t]+ToAc[t] 40 | 9 | 29.5 | 10.6 | 10 | 29.3 | 7 | 0.611 | 9 | 12 | 7 | 9 | 51.4 | 41.9 | 9 | 84.5 |  |
| *Retinoid[t]+H_2_O_2_[t] 34 | 10 | 29.3 | 10.5 | 11 | 28.4 | 11 | 0.599 | 13 | 11 | 11 | 10 | 51.6 | 41.9 | 10 | 84.9 |  |
| *Superoxidised solution[t] 10 | 11 | 28.5 | 14.1 | 12 | 27.4 | 12 | 0.596 | 12 | 14 | 12 | 11 | 52.4 | 42.7 | 11 | 86.5 |  |
| *Lincosamide[t]+Azelaic acid[t] 31 | 12 | 27.4 | 10.2 | 13 | 26.5 | 8 | 0.561 | 40 | 7 | 8 | 12 | 53.4 | 41.7 | 12 | 88.1 |  |
| *BP[t]+PhCh+PhTh 41 | 13 | 26.5 | 11.9 | 8 | 26.1 | 13 | 0.549 | 6 | 13 | 13 | 13 | 54.4 | 42.1 | 13 | 89.8 |  |
| PhCh [blue] 22 | 14 | 25.9 | 8.5 | 14 | 25.9 | 14 | 0.540 | 27 | 15 | 14 | 14 | 55.0 | 41.2 | 14 | 90.7 |  |
| BP[t]+Retinoid[t] 30 | 15 | 23.3 | 5.2 | 15 | 23.3 | 16 | 0.422 | 18 | 16 | 16 | 15 | 57.6 | 40.9 | 15 | 95.1 |  |
| *Azelaic acid[t]+Macrolide[t] 37 | 16 | 23.1 | 9.5 | 16 | 23.1 | 15 | 0.374 | 25 | 8 | 15 | 16 | 57.8 | 41.5 | 16 | 95.6 |  |
| Lincosamide[t]+Retinoid[t] 32 | 17 | 21.4 | 7.2 | 17 | 21.4 | 18 | 0.328 | 19 | 17 | 18 | 17 | 59.5 | 41.2 | 17 | 98.7 |  |
| *Macrolide[t]+Anti-fungal[t] 3 | 18 | 20.0 | 11.5 | 18 | 19.8 | 22 | 0.316 | 7 | 18 | 22 | 18 | 60.9 | 42.1 | 18 | 101.5 |  |
| *Retinoid[t]+ToAc[t]+PhCh[b&r] 39 | 19 | 17.5 | 13.3 | 20 | 17.1 | 17 | 0.307 | 21 | 20 | 17 | 19 | 63.4 | 42.5 | 19 | 106.2 |  |
| BP[t]+Macrolide[t] 29 | 20 | 17.3 | 9.8 | 19 | 16.9 | 19 | 0.284 | 16 | 19 | 19 | 20 | 63.6 | 41.7 | 20 | 106.3 |  |
| *Lincosamide[t]+ToAc[t] 36 | 21 | 15.8 | 11.8 | 23 | 15.4 | 20 | 0.215 | 29 | 21 | 20 | 21 | 65.1 | 42.2 | 22 | 108.6 |  |
| PhCh+PhTh 24 | 22 | 15.5 | 19.9 | 21 | 15.3 | 21 | 0.214 | 26 | 23 | 21 | 22 | 65.4 | 38.2 | 21 | 109.1 |  |
| Retinoid[t] 5 | 23 | 15.4 | 4.6 | 24 | 15.1 | 25 | 0.207 | 22 | 22 | 25 | 23 | 65.5 | 40.7 | 23 | 109.5 |  |
| BP[t]+Lincosamide[t] 28 | 24 | 15.1 | 5.5 | 22 | 13.0 | 27 | 0.200 | 32 | 24 | 27 | 24 | 65.8 | 40.9 | 24 | 110.1 |  |
| *Tetracycline[o]+Comb. PhPe[ph] 8 | 25 | 13.6 | 14.1 | 26 | 12.9 | 26 | 0.131 | 38 | 25 | 26 | 25 | 67.3 | 43.0 | 25 | 113.3 |  |
| Retinoid[t]+Macrolide[t] 35 | 26 | 13.4 | 10.4 | 28 | 12.8 | 29 | 0.110 | 14 | 27 | 29 | 26 | 67.5 | 41.9 | 26 | 113.5 |  |
| Comb. chemical peels[ph] 14 | 27 | 13.2 | 14.1 | 25 | 12.4 | 32 | 0.092 | 28 | 26 | 32 | 27 | 67.7 | 43.0 | 27 | 114.0 |  |
| Benzoyl peroxide[t] 3 | 28 | 12.8 | 5.4 | 27 | 11.9 | 40 | 0.057 | 37 | 28 | 40 | 28 | 68.1 | 40.8 | 28 | 114.3 |  |
| Antiseptics[t] 8 | 29 | 10.6 | 11.8 | 29 | 9.5 | 38 | 0.045 | 23 | 29 | 38 | 29 | 70.3 | 42.1 | 29 | 118.6 |  |
| Topical acid[t] 12 | 30 | 9.3 | 8.5 | 30 | 8.8 | 36 | 0.038 | 35 | 32 | 36 | 30 | 71.6 | 41.6 | 30 | 121.0 |  |
| Macrolide[t] 7 | 31 | 8.9 | 5.7 | 31 | 8.7 | 24 | 0.036 | 31 | 30 | 24 | 31 | 72.0 | 40.9 | 31 | 121.6 |  |
| Retinoid tcd<120mg/kg(sc)[o] 16 | 32 | 8.8 | 12.3 | 32 | 7.1 | 30 | 0.035 | 39 | 31 | 30 | 32 | 72.1 | 41.9 | 32 | 122.0 |  |
| Co-cyprindiol[o] 19 | 33 | 7.6 | 8.1 | 34 | 7.0 | 23 | 0.019 | 30 | 33 | 23 | 33 | 73.3 | 41.4 | 33 | 124.2 |  |
| Comb. Oral Contraceptive[o] 20 | 34 | 7.3 | 5.7 | 33 | 6.8 | 33 | 0.016 | 36 | 34 | 33 | 34 | 73.6 | 41.0 | 34 | 124.7 |  |
| Azelaic acid[t] 6 | 35 | 6.8 | 6.3 | 35 | 6.4 | 28 | 0.012 | 33 | 36 | 28 | 35 | 74.1 | 40.8 | 35 | 125.5 |  |
| Tetracycline[o] 17 | 36 | 6.5 | 10.4 | 36 | 4.9 | 31 | 0.003 | 34 | 35 | 31 | 36 | 74.3 | 41.9 | 36 | 126.2 |  |
| Lincosamide[t] 4 | 37 | 3.4 | 4.6 | 37 | 2.8 | 35 | 0.002 | 24 | 38 | 35 | 37 | 77.5 | 40.8 | 37 | 131.9 |  |
| Macrolide[o] 18 | 38 | 0.7 | 14.4 | 38 | -4.7 | 39 | 0.002 | 20 | 37 | 39 | 38 | 80.2 | 43.0 | 38 | 137.5 |  |
| Placebo 1 | - | 0(R) | - | - | - | - | - | - | - | - | 39 | 80.9 | 40.7 | 39 | 138.5 |  |
| Fusidic acid[t] 9 | 39 | -2.5 | 8.8 | 39 | -7.4 | 34 | 0.001 | 15 | 40 | 34 | 40 | 83.4 | 41.6 | 40 | 143.5 |  |
| *Anti-fungal[t] 11 | 40 | -10.0 | 22.3 | 40 | -24.7 | 37 | 0.000 | 17 | 39 | 37 | 41 | 90.9 | 46.1 | 41 | 158.5 |  |

Abbreviations: BP Benzoyl peroxide; tcd total cumulative dose; [t] topical; [o] oral; [ph] physical; (sc) single course; ToAc Topical Acid; PhCh Photochemical

therapy; PhTh Photothermal therapy; PhDy Photodynamic therapy; Comb. Combined; Rk rank (values are ranks on EV); R Reference Treatment 1.

***Mild-to-Moderate Acne***

***Type of model****:* Random study effects, fixed class effect model with 72 treatments in 40 classes relative to placebo, and bias adjustment for small study effects.

***Outcome*:** % Change from Baseline.

***MCID***: 25%, ***GRADE probability cutoff****:* 0.975

***EV and LaEV Decision rules***

*Stage 1.*  38 treatment classes have positive EV, and 37 have positive LaEV

*Stage 2.* *EV*: Only 2 further treatments besides the best treatment are recommended, because they are not worse than the best treatment by more than the MCID. On *LaEV* only one further treatment is recommended

***GRADE***

*GRADE:* Only one treatment is promoted to Category 1, ranked 2^nd^ on EV, and therefore this treatment is the only one to be included in the final recommendation

***RANKING SYSTEMS***: Pr(V>T) and Pr(Best) are erratic, respectively privileging less uncertain and more uncertain treatments. However, both Pr(Best) and SUCRA pick out the same top 3 treatments as EV; Pr(V>T) 2 of the 3.

***Comments:*** A number of treatments (marked with an asterisk in Table S2) were excluded from consideration by the guideline development committee on the grounds of small sample size. Two of them would have been recommended by EV. One was excluded by LaEV at Stage 2.

**Fig S2** Mild to Moderate Acne


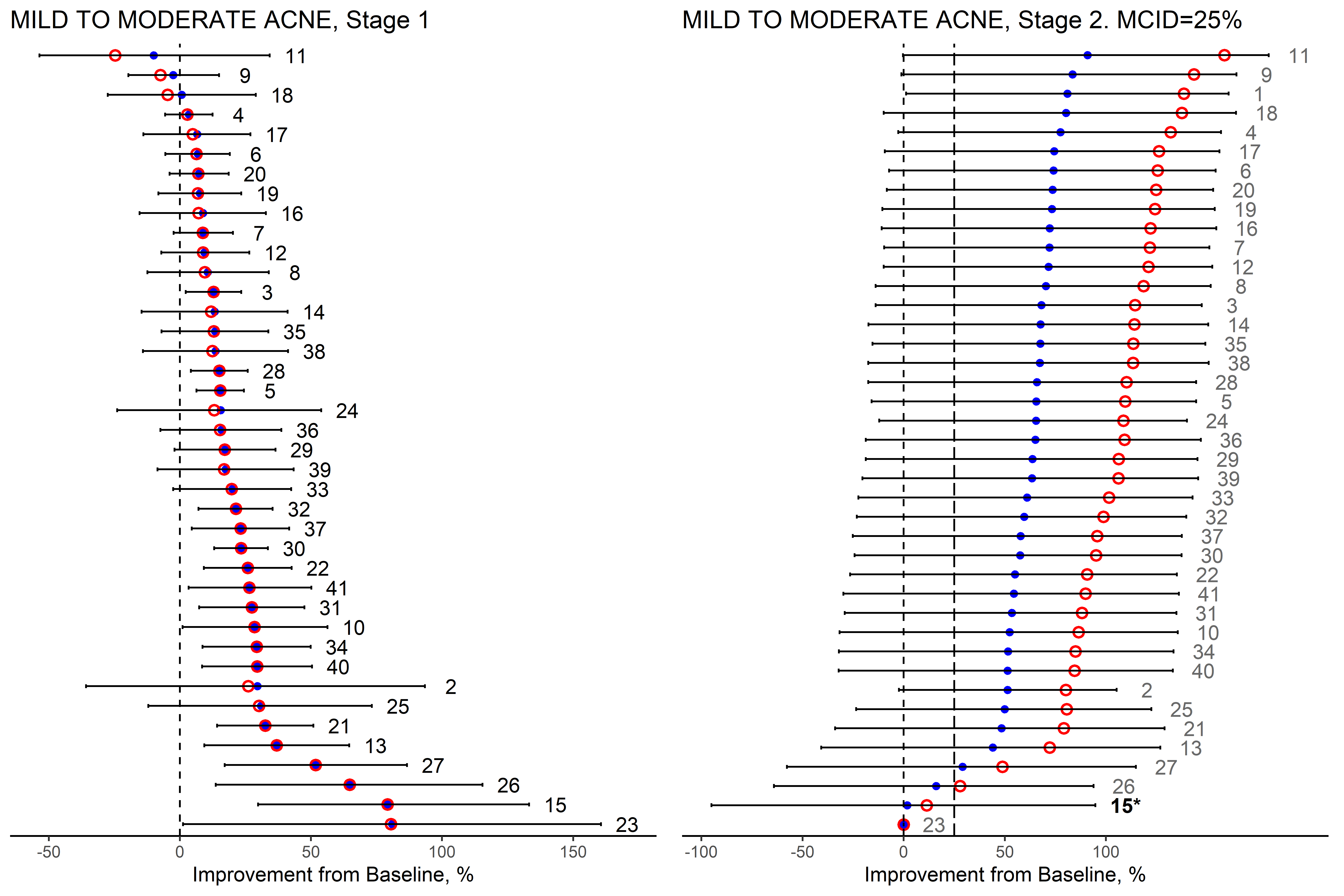


**A3. Moderate to Severe Depression**

**Table S3.**

| **Treatment**  (numbering as in NICE guidelines) | **STAGE 1**  **Decision Rules** | | | | | | | **Ranking systems** | | | **STAGE 2**  **Decision Rules** | | | | | **FINAL**  **GRADE**  (0.85)  Category 1 |
| --- | --- | --- | --- | --- | --- | --- | --- | --- | --- | --- | --- | --- | --- | --- | --- | --- |
|  | **EV** | | | **LaEV** | | **GRADE**  (0.85)  Category 1 | |  |  |  | **EV** | | | **LaEV** | |  |
|  | **Rk** | **EV** | **Sd** | **Rk** | **LaEV** | **Rk** | **Pr(V>T)** | **P(Best)** | **SUCRA** | **Pr(V>T)** | **Rk** | **EV** | **sd** | **Rk** | **LaEV** |  |
| Exercise group + AD 17 | 1 | 1.37 | 0.70 | 1 | 1.36 | 3 | 0.964 | 1 | 1 | 3 | 1(R) | 0.00 | 0.00 | 1 | 0.00 | 3 |
| CT & CBT group + AD 25 | 2 | 1.23 | 0.83 | 2 | 1.20 | 9 | 0.959 | 2 | 3 | 9 | 2 | 0.14 | 1.08 | 3 | 0.36 | 9 |
| CT & CBT individual + AD 6 | 3 | 1.18 | 0.41 | 3 | 1.18 | 1 | 0.903 | 4 | 2 | 1 | 3 | 0.18 | 0.81 | 2 | 0.40 | 1 |
| Yoga group 10 | 4 | 1.05 | 0.61 | 4 | 1.04 | 10 | 0.883 | 3 | 4 | 10 | 4 | 0.31 | 0.92 | 4 | 0.59 | 10 |
| Self-help 21 | 5 | 0.98 | 0.69 | 5 | 0.95 | 6 | 0.857 | 5 | 5 | 6 | 5 | 0.39 | 0.98 | 5 | 0.70 | 6 |
| BT individual 8 | 6 | 0.86 | 0.38 | 6 | 0.86 | 7 | 0.856 | 20 | 6 | 7 | 6 | 0.50 | 0.79 | 6 | 0.81 | 7 |
| Light therapy + AD 11 | 7 | 0.86 | 0.37 | 7 | 0.86 | 5 | 0.847 | 26 | 7 | 5 | 7 | 0.51 | 0.78 | 7 | 0.82 |  |
| Problem solving individual 15 | 8 | 0.86 | 0.44 | 8 | 0.85 | 2 | 0.842 | 12 | 8 | 2 | 8 | 0.51 | 0.82 | 8 | 0.84 |  |
| Acupuncture + AD 39 | 9 | 0.78 | 0.18 | 9 | 0.78 | 8 | 0.832 | 8 | 9 | 8 | 9 | 0.58 | 0.72 | 9 | 0.90 |  |
| CT & CBT individual 13 | 10 | 0.78 | 0.27 | 10 | 0.78 | 4 | 0.832 | 6 | 10 | 4 | 10 | 0.59 | 0.75 | 10 | 0.93 |  |
| Counselling individual 14 | 11 | 0.67 | 0.41 | 11 | 0.66 | 11 | 0.699 | 7 | 11 | 11 | 11 | 0.70 | 0.81 | 11 | 1.12 |  |
| IPT individual+ AD 22 | 12 | 0.66 | 0.65 | 12 | 0.61 | 13 | 0.634 | 13 | 12 | 13 | 12 | 0.71 | 0.95 | 12 | 1.19 |  |
| Self-help with support 19 | 13 | 0.60 | 0.58 | 14 | 0.57 | 12 | 0.598 | 24 | 13 | 12 | 13 | 0.76 | 0.91 | 13 | 1.25 |  |
| ST PDT individual 18 | 14 | 0.58 | 0.36 | 13 | 0.56 | 14 | 0.584 | 11 | 14 | 14 | 14 | 0.79 | 0.78 | 14 | 1.26 |  |
| IPT individual 16 | 15 | 0.45 | 0.46 | 15 | 0.41 | 15 | 0.450 | 23 | 15 | 15 | 15 | 0.92 | 0.83 | 15 | 1.50 |  |
| Acupuncture 24 | 16 | 0.40 | 0.31 | 16 | 0.39 | 19 | 0.415 | 15 | 20 | 20 | 16 | 0.96 | 0.76 | 16 | 1.55 |  |
| Mirtazapine 23 | 17 | 0.35 | 0.07 | 17 | 0.35 | 16 | 0.338 | 10 | 16 | 16 | 17 | 1.01 | 0.70 | 17 | 1.62 |  |
| SNRIs 4 | 18 | 0.32 | 0.05 | 18 | 0.32 | 26 | 0.266 | 14 | 17 | 26 | 18 | 1.04 | 0.70 | 18 | 1.67 |  |
| TCAs 3 | 19 | 0.30 | 0.99 | 20 | 0.29 | 21 | 0.232 | 21 | 18 | 21 | 19 | 1.06 | 1.21 | 20 | 1.74 |  |
| ST PDP individual+AD 2 | 20 | 0.29 | 0.12 | 22 | 0.24 | 23 | 0.195 | 9 | 21 | 23 | 20 | 1.08 | 0.70 | 22 | 1.82 |  |
| CT & CBT group 12 | 21 | 0.25 | 0.42 | 21 | 0.19 | 24 | 0.147 | 16 | 19 | 24 | 21 | 1.11 | 0.81 | 21 | 1.83 |  |
| SSRIs 26 | 22 | 0.24 | 0.04 | 25 | 0.12 | 20 | 0.024 | 19 | 23 | 19 | 22 | 1.13 | 0.69 | 19 | 1.87 |  |
| Exercise group 20 | 23 | 0.20 | 0.56 | 23 | 0.10 | 17 | 0.015 | 17 | 26 | 17 | 23 | 1.17 | 0.89 | 23 | 1.95 |  |
| Exercise individual 1 | 24 | 0.14 | 0.61 | 19 | 0.05 | 18 | 0.003 | 25 | 24 | 18 | 24 | 1.23 | 0.92 | 25 | 2.03 |  |
| Trazodone 9 | 25 | 0.13 | 0.08 | 24 | 0.01 | 22 | 0.000 | 22 | 22 | 22 | 25 | 1.24 | 0.70 | 24 | 2.07 |  |
| Placebo 27 | - | 0(R) | - | - | 0(R) | - | - | - | - | - | 26 | 1.37 | 0.70 | 26 | 2.27 |  |
| Individual Counselling+AD 7 | 26 | -0.29 | 1.32 | 26 | -0.96 | 25 | 0.000 | 18 | 25 | 25 | 27 | 1.65 | 1.49 | 27 | 2.99 |  |

Abbreviations: R=reference treatment. Rk EV Treatment ranking; AD anti-depressants; CT Cognitive therapy; CBT Cognitive Behavioural therapy; ST Short term; SSRI Selective serotonin reuptake inhibitors ; SNRI Serotonin and norepinephrine reuptake inhibitors ; TCA Tricyclic antidepressant; IPT Interpersonal Psychotherapy; BT Behavioural therapy; PDT Psychodynamic psychotherapy; R Reference Treatment 1.

***Moderate to severe depression***^2^

***Type of model****:* Random study and random class effects model with 99 treatments in 50 treatment classes, relative to placebo, bias-adjusted for small study effects. The committee excluded treatments from the decision set to which less than 50 patients had been randomized, and other treatments not available in the UK. The decision set consisted of 26 classes of treatment relative to Pill Placebo.

***Outcome*:** Standardized Mean Difference

***MCID***: 0.50 ***GRADE probability cutoff****:* 0.85

***EV and LaEV Decision rules***

*Stage 1.* 26 treatment classes had a positive EV; the same 26 treatment classes have positive LaEV

*Stage 2.* On EV 5 treatments are recommended including the best treatment as they were not worse than the best treatment by more than the MCID. On LaEV, only three are recommended.

***Ranking Systems***: Pr(V>T) ranks treatments 3,9,10 especially highly as these have exceptionally low SD. SUCRA ranking was close to EV; Pr(Best) was highly erratic. However, both Pr(Best) and SUCRA pick out the same 5 top-ranked treatments as EV.

***GRADE:*** No treatments were better than Pill Placebo by 0.50 with probability > 0.975. For purposes of illustration a lower threshold, 0.85, was selected. On this basis 6 treatments were promoted to Category 1, included those ranked 9^th^ and 10^th^ on EV. As none of these Category 1 treatments was superior to another by the MCID with probability > 0.85, none were promoted to Category 2, and all 6 are recommended.

**Figure S3** Moderate to severe depression


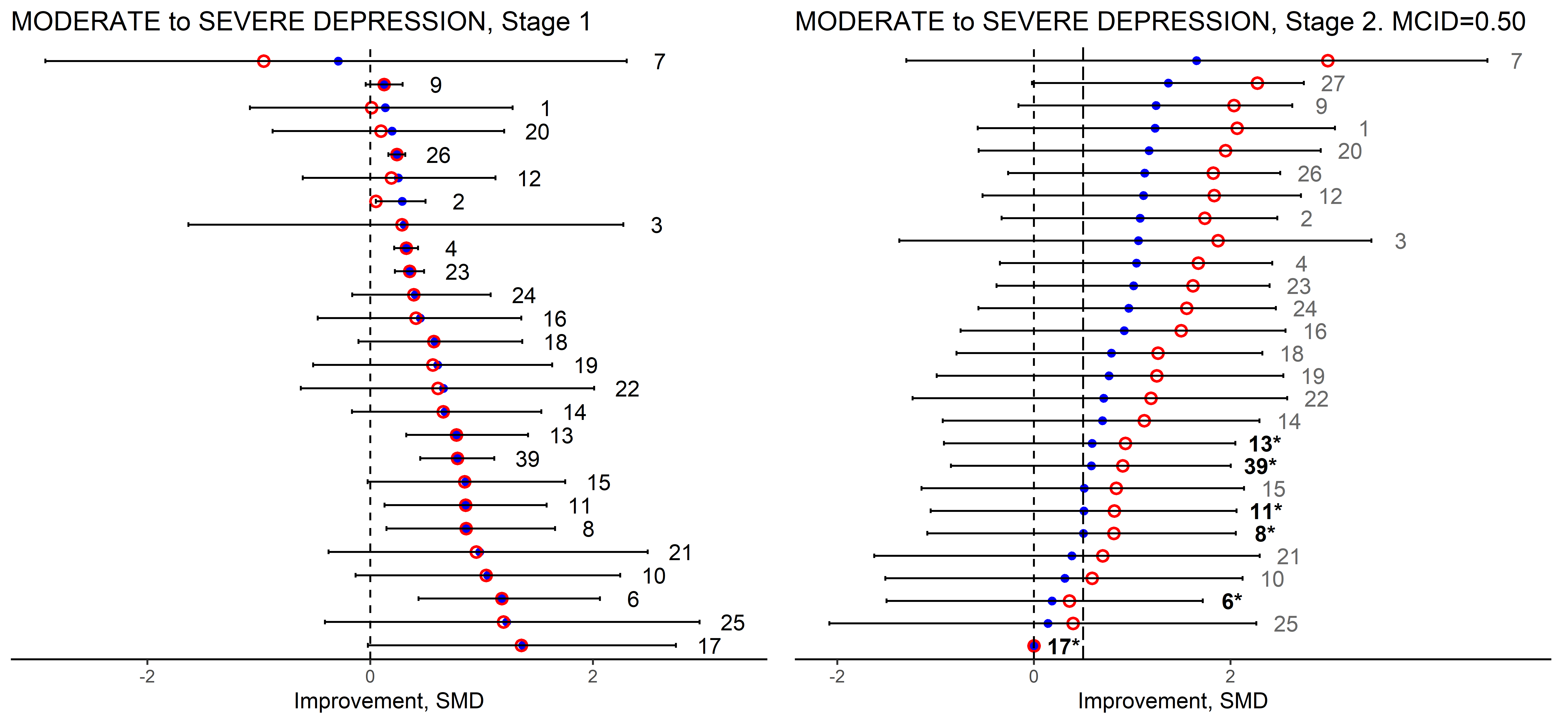


**A4. Tranexamic Acid (TXA) for primary joint replacement**.^3^

**Table S4.** Outcome is reduced risk of transfusion, %, relative to Intra-articular administration

| **Treatment**  (numbering as in NICE guidelines) | **STAGE 1**  **Decision Rules** | | | | | | | **Ranking systems** | | | **STAGE 2**  **Decision Rules** | | | | | **FINAL**  **GRADE**  (0.975)  Category 1 |
| --- | --- | --- | --- | --- | --- | --- | --- | --- | --- | --- | --- | --- | --- | --- | --- | --- |
|  | **EV** | | | **LaEV** | | **GRADE**  (0.975)  Category 1 | |  |  |  | **EV** | | | **LaEV** | |  |
|  | **Rk** | **EV** | **Sd** | **Rk** | **LaEV** | **Rk** | **Pr(V>T)** | **P(Best)** | **SUCRA** | **Pr(V>T)** | **Rk** | **EV** | **Sd** | **Rk** | **LaEV** |  |
| Intra-articular and oral (5) | 1 | 6.48 | 4.49 | 1 | 6.33 | 2 | 0.987 | 1 | 1 | 2 | 1 | 0.00 | 0.00 | 1 | 0.00 | 2 |
| Intra-articular & Intravenous (4) | 2 | 5.50 | 2.99 | 2 | 5.50 | 1 | 0.933 | 2 | 2 | 1 | 2 | 0.97 | 3.26 | 2 | 1.42 |  |
| Oral (3) | 3 | 1.11 | 1.87 | 3 | 0.84 | 3 | 0.169 | 3 | 3 | 3 | 3 | 5.37 | 4.48 | 3 | 8.97 |  |
| Intravenous (2) | 4 | 0.55 | 0.99 | 4 | 0.40 | 4 | 0.003 | 4 | 4 | 4 | 4 | 5.93 | 4.40 | 4 | 10.00 |  |
| Intra-articular (1) | - | 0(R) | - | - | 0(R) | - | - | - | - | - | 5 | 6.48 | 4.49 | 5 | 11.05 |  |

**Tranexamic Acid (TXA) for primary joint replacement**.

***Type of model****:* Fixed study, fixed class model with 4 classes of tranexamic acid (TXA) administration relative to Intra-articular administration

***Outcome*:** Reduction in risk of transfusion, expressed as %.

***MCID***: RR=1.50. ***GRADE probability cutoff****:* 0.975

***EV and LaEV Decision rules***

*Stage 1.* All 4 treatments are superior to the reference treatment, on both EV and LaEV criteria.

*Stage 2*. Besides the best treatment, one other treatment is within an MCID of the best treatment. Both are therefore recommended.

***GRADE.*** Only one treatment, ranked 2^nd^ on EV, was promoted to Category 1, and was therefore recommended.

***Ranking Systems***: Pr(best) and SUCRA produced the same ranking as EV. The highest ranked treatment on Pr(V>T) was ranked 2^nd^ on EV

**Figure S4.** Tranexamic Acid in Joint Replacement.


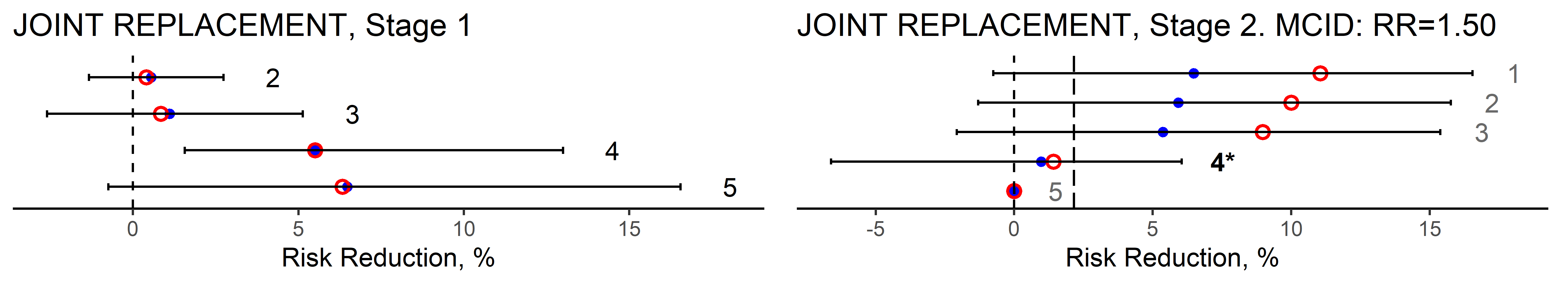


**A5. Headache Prophylaxis.**

**Table S5.** Headache prophylaxis. Outcome is reduction in headache-days per month relative to placebo .

| **Treatment**  (numbering as in NICE guidelines) | **STAGE 1**  **Decision Rules** | | | | | | | **Ranking systems** | | | **STAGE 2**  **Decision Rules** | | | | | **FINAL**  **GRADE**  (0.975)  Category 1 |
| --- | --- | --- | --- | --- | --- | --- | --- | --- | --- | --- | --- | --- | --- | --- | --- | --- |
|  | **EV** | | | **LaEV** | | **GRADE**  (0.975)  Category 1 | |  |  |  | **EV** | | | **LaEV** | |  |
|  | **Rk** | **EV** | **Sd** | **Rk** | **LaEV** | **Rk** | **Pr(V>T)** | **P(Best)** | **SUCRA** | **Pr(V>T)** | **Rk** | **EV** | **sd** | **Rk** | **LaEV** |  |
| Propranolol (6) | 1 | 1.19 | 0.50 | 1 | 1.18 | 3 | 0.988 | 1 | 1 | 3 | 1 | 0.00 | 0.00 | 1 | 0.00 | 3 |
| Amitriptyline (2) | 2 | 1.14 | 0.66 | 2 | 1.13 | 1 | 0.925 | 2 | 2 | 1 | 2 | 0.05 | 0.78 | 2 | 0.18 |  |
| Topiramate (5) | 3 | 1.04 | 0.24 | 3 | 1.04 | 2 | 0.847 | 5 | 3 | 2 | 3 | 0.15 | 0.48 | 3 | 0.21 |  |
| Propranolol & Nadolol (7) | 4 | 0.60 | 0.52 | 4 | 0.57 | 4 | 0.591 | 3 | 4 | 4 | 4 | 0.59 | 0.72 | 4 | 0.91 |  |
| Telmisartan (1) | 5 | 0.51 | 0.92 | 5 | 0.35 | 5 | 0.504 | 4 | 5 | 5 | 5 | 0.68 | 1.04 | 5 | 1.18 |  |
| Gabapentin (4) | 6 | 0.00 | 0.81 | 6 | -0.32 | 6 | 0.264 | 6 | 6 | 6 | 6 | 1.19 | 0.95 | 7 | 1.90 |  |
| Placebo (1) | - | 0(R) | - | - | 0(R) | - | - | - | - | - | 7 | 1.19 | 0.50 | 6 | 2.00 |  |
| Divalproex Sodium (3) | 7 | -0.12 | 0.56 | 7 | -0.40 | 7 | 0.129 | 7 | 7 | 7 | 8 | 1.31 | 0.75 | 8 | 2.17 |  |

R Reference Treatment 1.

**Headache prophylaxis**.

***Type of model****:* Random effects model of 7 treatments relative to placebo

***Outcome*:** Reduction in hospital-days per month

***MCID***: 0.5 days. ***GRADE probability cutoff****:* 0.975

***EV and LaEV Decision rules***

*Stage 1.*  6 treatments are superior to the placebo on EV and 5 on LaEV.

*Stage 2.* Two treatments were recommended alongside the best treatment; and decisions based on EV and LaEV were identical.

***GRADE:*** Only one treatment, the one ranked 3^rd^ on EV, was promoted to Category 1, and was therefore recommended.

***Ranking Systems:*** The SUCRA ranking was the same as the EV ranking. With Pr(Best) the treatment ranked 5^th^ by EV was ranked 3^rd^. On Pr(V>T) the best treatment was ranked 3^rd^ by EV

**Figure S5. Headache prophylaxis**

**
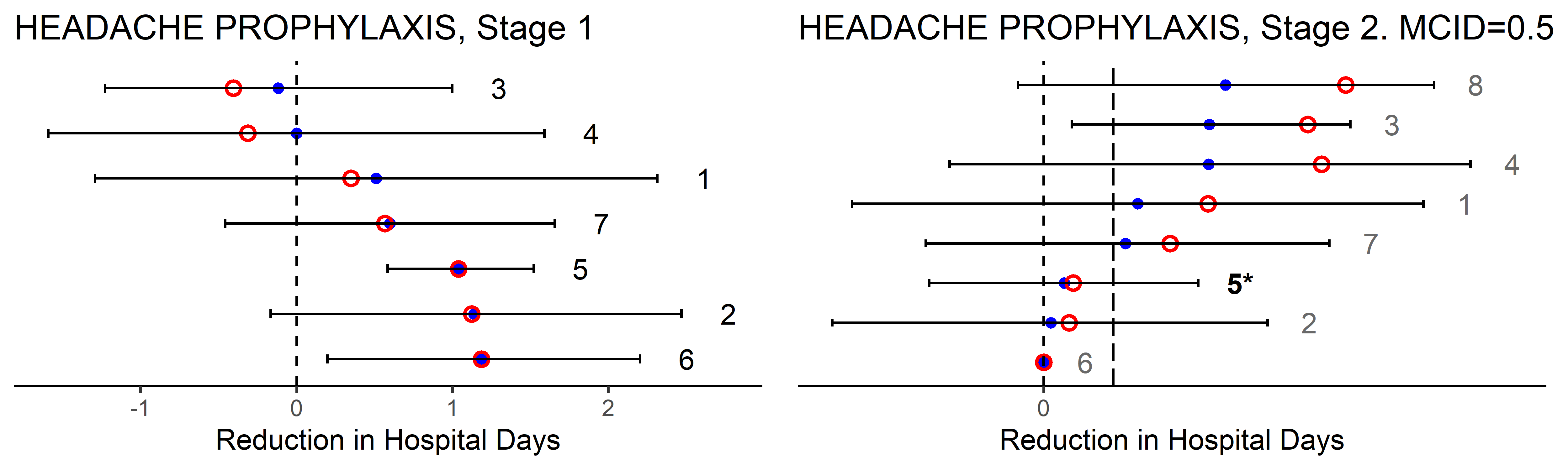
**

**A6: Social Anxiety (Treatment)** ^4^

**Table S6.** Nice Guideline Social Anxiety. Outcome is improvement in clinical score, on SMD scale, relative to waitlist.

| **Treatment**  (numbering as in NICE guidelines) | **STAGE 1**  **Decision Rules** | | | | | | | **Ranking systems** | | | **STAGE 2**  **Decision Rules** | | | | | **FINAL**  **GRADE**  (0.975)  Category 1 |
| --- | --- | --- | --- | --- | --- | --- | --- | --- | --- | --- | --- | --- | --- | --- | --- | --- |
|  | **EV** | | | **LaEV** | | **GRADE**  (0.975)  Category 1 | |  |  |  | **EV** | | | **LaEV** | |  |
|  | **Rk** | **EV** | **Sd** | **Rk** | **LaEV** | **Rk** | **Pr(V>T)** | **P(Best)** | **SUCRA** | **Pr(V>T)** | **Rk** | **EV** | **sd** | **Rk** | **LaEV** |  |
| CBT group Heim + Phenelzine 40 | 1 | 1.68 | 0.21 | 1 | 1.68 | 19 | 1.000 | 1 | 1 | 19 | 1 | 0.00 | 0.00 | 1 | 0.00 | 19 |
| Cognitive therapy 35 | 2 | 1.56 | 0.15 | 2 | 1.56 | 5 | 1.000 | 2 | 2 | 5 | 2 | 0.12 | 0.24 | 2 | 0.13 | 5 |
| Paroxetine + Clonazapam 38 | 3 | 1.35 | 0.29 | 3 | 1.35 | 7 | 1.000 | 3 | 5 | 7 | 3 | 0.33 | 0.31 | 3 | 0.39 | 7 |
| Psychodynamic + Clonazepam 37 | 4 | 1.28 | 0.27 | 4 | 1.28 | 2 | 1.000 | 4 | 3 | 2 | 4 | 0.40 | 0.30 | 5 | 0.45 | 2 |
| Phenelzine 22 | 5 | 1.27 | 0.15 | 5 | 1.27 | 1 | 1.000 | 6 | 4 | 1 | 5 | 0.41 | 0.20 | 4 | 0.48 | 1 |
| CBT individual + Moclobemide 39 | 6 | 1.23 | 0.25 | 6 | 1.23 | 11 | 1.000 | 28 | 7 | 11 | 6 | 0.45 | 0.29 | 6 | 0.54 | 11 |
| CBT individual 34 | 7 | 1.19 | 0.15 | 7 | 1.19 | 22 | 1.000 | 8 | 6 | 22 | 7 | 0.50 | 0.24 | 7 | 0.59 | 22 |
| CBT group enhanced exposure 31 | 8 | 1.10 | 0.20 | 8 | 1.10 | 12 | 1.000 | 30 | 8 | 12 | 8 | 0.58 | 0.27 | 8 | 0.74 | 12 |
| Clonazapam 20 | 9 | 1.07 | 0.19 | 9 | 1.07 | 13 | 0.999 | 5 | 9 | 13 | 9 | 0.61 | 0.25 | 9 | 0.78 | 13 |
| CBT individual Heimberg 33 | 10 | 1.02 | 0.20 | 10 | 1.02 | 9 | 0.999 | 10 | 11 | 9 | 10 | 0.66 | 0.29 | 10 | 0.87 | 8 |
| Paroxetine 17 | 11 | 0.99 | 0.14 | 11 | 0.99 | 8 | 0.999 | 7 | 10 | 8 | 11 | 0.69 | 0.21 | 11 | 0.90 | 9 |
| CT abbreviated experiments 32 | 12 | 0.97 | 0.12 | 12 | 0.97 | 3 | 0.998 | 18 | 12 | 3 | 12 | 0.71 | 0.23 | 12 | 0.95 | 3 |
| Venlafaxine <75 18 | 13 | 0.96 | 0.15 | 13 | 0.96 | 6 | 0.998 | 17 | 13 | 6 | 13 | 0.72 | 0.21 | 13 | 0.96 | 6 |
| CBT group + Fluoxetine 36 | 14 | 0.95 | 0.19 | 14 | 0.95 | 4 | 0.998 | 23 | 14 | 4 | 14 | 0.73 | 0.26 | 14 | 0.98 | 4 |
| Fluvoxamine 16 | 15 | 0.94 | 0.16 | 15 | 0.94 | 25 | 0.997 | 9 | 15 | 25 | 15 | 0.75 | 0.22 | 15 | 1.01 | 25 |
| Sertraline 12 | 16 | 0.91 | 0.16 | 16 | 0.91 | 15 | 0.997 | 39 | 16 | 15 | 16 | 0.77 | 0.22 | 16 | 1.05 | 15 |
| Gabapentin 10 | 17 | 0.89 | 0.27 | 17 | 0.89 | 27 | 0.996 | 14 | 17 | 27 | 17 | 0.80 | 0.31 | 17 | 1.12 | 27 |
| Social skills training 24 | 18 | 0.88 | 0.25 | 18 | 0.88 | 29 | 0.996 | 26 | 19 | 29 | 18 | 0.80 | 0.32 | 19 | 1.12 | 29 |
| Self-help internet with support 7 | 19 | 0.88 | 0.08 | 19 | 0.88 | 16 | 0.995 | 24 | 18 | 16 | 19 | 0.81 | 0.22 | 20 | 1.13 | 16 |
| Escitalopram 14 | 20 | 0.87 | 0.16 | 20 | 0.87 | 10 | 0.994 | 12 | 20 | 10 | 20 | 0.81 | 0.23 | 18 | 1.14 | 10 |
| Fluoxetine 15 | 21 | 0.87 | 0.15 | 21 | 0.87 | 21 | 0.992 | 36 | 21 | 21 | 21 | 0.82 | 0.23 | 21 | 1.14 | 21 |
| CBT group 29 | 22 | 0.85 | 0.09 | 22 | 0.85 | 14 | 0.990 | 34 | 23 | 14 | 22 | 0.83 | 0.21 | 22 | 1.16 | 14 |
| Alprazolam 19 | 23 | 0.85 | 0.28 | 24 | 0.85 | 20 | 0.990 | 25 | 24 | 20 | 23 | 0.83 | 0.32 | 24 | 1.18 | 20 |
| Self-help book with support 6 | 24 | 0.85 | 0.16 | 23 | 0.85 | 24 | 0.983 | 33 | 22 | 24 | 24 | 0.83 | 0.26 | 23 | 1.19 | 24 |
| Self-help book no support 4 | 25 | 0.84 | 0.12 | 25 | 0.84 | 31 | 0.943 | 19 | 25 | 31 | 25 | 0.84 | 0.24 | 25 | 1.19 |  |
| Citalopram 13 | 26 | 0.83 | 0.23 | 26 | 0.83 | 18 | 0.932 | 32 | 28 | 18 | 26 | 0.85 | 0.28 | 26 | 1.21 |  |
| Exposure in vivo 23 | 27 | 0.83 | 0.12 | 27 | 0.83 | 26 | 0.930 | 16 | 26 | 26 | 27 | 0.85 | 0.23 | 27 | 1.22 |  |
| Levetiracetam 9 | 28 | 0.82 | 0.33 | 28 | 0.82 | 17 | 0.928 | 20 | 27 | 17 | 28 | 0.86 | 0.37 | 28 | 1.25 |  |
| CBT group Heimberg 30 | 29 | 0.80 | 0.11 | 29 | 0.80 | 23 | 0.895 | 21 | 30 | 23 | 29 | 0.88 | 0.20 | 29 | 1.27 |  |
| Mirtazapine 11 | 30 | 0.80 | 0.33 | 30 | 0.80 | 32 | 0.890 | 15 | 29 | 32 | 30 | 0.88 | 0.36 | 30 | 1.30 |  |
| Moclobemide 21 | 31 | 0.73 | 0.15 | 31 | 0.73 | 33 | 0.879 | 11 | 31 | 33 | 31 | 0.95 | 0.22 | 31 | 1.40 |  |
| Pregabalin 400 8 | 32 | 0.72 | 0.18 | 32 | 0.72 | 28 | 0.838 | 13 | 32 | 28 | 32 | 0.96 | 0.24 | 32 | 1.43 |  |
| Self-help internet no support 5 | 33 | 0.66 | 0.14 | 33 | 0.66 | 34 | 0.823 | 31 | 33 | 34 | 33 | 1.02 | 0.25 | 33 | 1.54 |  |
| Attention-matched control 2 | 34 | 0.63 | 0.14 | 34 | 0.63 | 30 | 0.819 | 27 | 35 | 30 | 34 | 1.05 | 0.23 | 34 | 1.61 |  |
| Psychodynamic psychotherapy 28 | 35 | 0.62 | 0.16 | 35 | 0.62 | 35 | 0.778 | 40 | 34 | 35 | 35 | 1.06 | 0.25 | 35 | 1.63 |  |
| Pill Placebo 1 | 36 | 0.47 | 0.12 | 36 | 0.47 | 36 | 0.389 | 38 | 39 | 36 | 36 | 1.22 | 0.20 | 36 | 1.93 |  |
| Interpersonal psychotherapy 27 | 37 | 0.43 | 0.20 | 37 | 0.43 | 37 | 0.365 | 37 | 37 | 37 | 37 | 1.25 | 0.28 | 37 | 2.00 |  |
| Mindfulness cognitive therapy 26 | 38 | 0.39 | 0.22 | 38 | 0.39 | 39 | 0.338 | 35 | 36 | 39 | 38 | 1.29 | 0.28 | 38 | 2.08 |  |
| Exercise 3 | 39 | 0.35 | 0.37 | 39 | 0.31 | 38 | 0.309 | 22 | 38 | 38 | 39 | 1.33 | 0.41 | 39 | 2.17 |  |
| Supportive therapy 25 | 40 | 0.26 | 0.24 | 40 | 0.24 | 40 | 0.152 | 29 | 40 | 40 | 40 | 1.42 | 0.30 | 40 | 2.35 |  |
| Waitlist control 0 | 41 | 0(R) | - | - | 0(R) | - | - | - | - | - | 41 | 1.68 | 0.21 | 41 | 2.86 |  |

R Reference Treatment 1.

**Social Anxiety (Treatment)**

***Type of model****:* Random study effects, random class model (17 classes), with 40 individual treatments relative to waitlist control forming the decision set.^4,5^

***Outcome*:** Standardized Mean Difference

***MCID***: 0.50 ***GRADE probability cutoff****:* 0.975

***EV and LaEV Decision rules***

*Stage 1.* All 40 treatments were superior to waitlist control on EV and LaEV

Stage 2. On EV, a total of 7 treatments were recommended, all those within 0.50 of the best treatment. Only the highest ranked 5 of these treatments were recommended on LaEV

***GRADE****.* 24 treatments were promoted to Category 1, but none of them were superior to any other by 0.50 with probability> 0.975, so all 24 were recommended.

***Ranking Systems****:* SUCRA picks out the same top 7 treatments as EV. Pr(V>T) accords the highest rank to the treatment ranked 19^th^ by EV and LaEV, due to its exceptionally low SD. Pr(Best) places the treatments ranked 28^th^ by EV and LaEV in the top 7 treatments, due to its exceptionally high SD.

**Figure S6.**  Social Anxiety (Treatment).


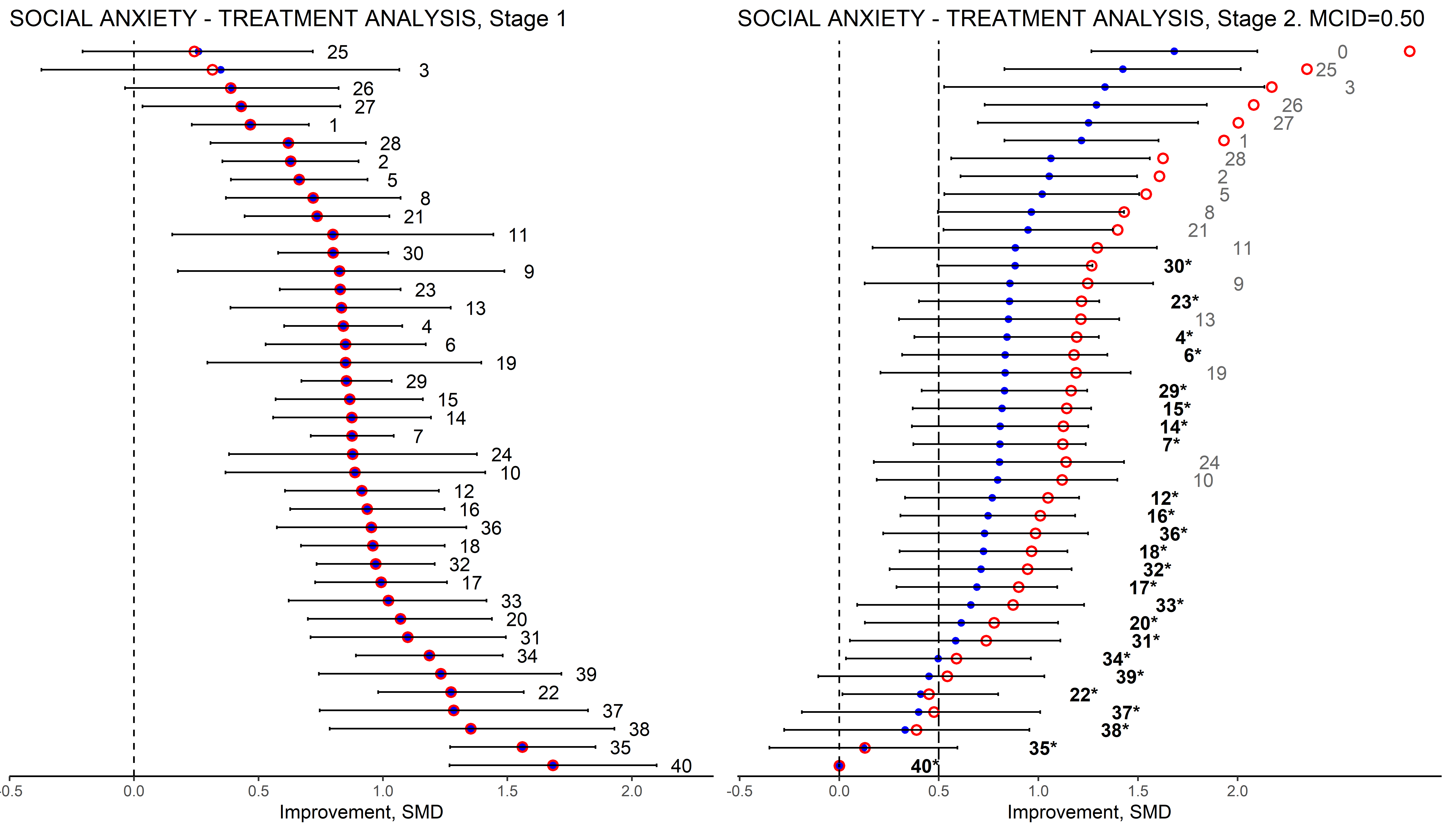


**A7. Social Anxiety, Class analysis**^6^

**Table S7.** Social Anxiety, class analysis. . Outcome is improvement in clinical score, on SMD scale, relative to waitlist.

| **Treatment**  (numbering as in NICE guidelines) | **STAGE 1**  **Decision Rules** | | | | | | | **Ranking systems** | | | **STAGE 2**  **Decision Rules** | | | | | **FINAL**  **GRADE**  (0.975)  Category 1 |
| --- | --- | --- | --- | --- | --- | --- | --- | --- | --- | --- | --- | --- | --- | --- | --- | --- |
|  | **EV** | | | **LaEV** | | **GRADE**  (0.975)  Category 1 | |  |  |  | **EV** | | | **LaEV** | |  |
|  | **Rk** | **EV** | **Sd** | **Rk** | **LaEV** | **Rk** | **Pr(V>T)** | **P(Best)** | **SUCRA** | **Pr(V>T)** | **Rk** | **EV** | **sd** | **Rk** | **LaEV** |  |
| Combined 17 | 1 | 1.30 | 0.22 | 1 | 1.30 | 1 | 1.000 | 1 | 1 | 1 | 1 | 0.00 | 0.00 | 1 | 0.00 | 1 |
| CBT individual 16 | 2 | 1.19 | 0.19 | 2 | 1.19 | 2 | 0.999 | 2 | 2 | 2 | 2 | 0.12 | 0.27 | 2 | 0.13 | 2 |
| MAOI 11 | 3 | 1.00 | 0.28 | 3 | 1.00 | 6 | 0.995 | 3 | 3 | 6 | 3 | 0.30 | 0.33 | 3 | 0.44 | 6 |
| Benzodiazepines 10 | 4 | 0.96 | 0.31 | 4 | 0.96 | 5 | 0.977 | 10 | 4 | 5 | 4 | 0.34 | 0.34 | 4 | 0.45 | 5 |
| CBT group 15 | 5 | 0.92 | 0.21 | 5 | 0.92 | 3 | 0.963 | 4 | 5 | 3 | 5 | 0.38 | 0.28 | 6 | 0.88 |  |
| SSRI/SNRI 9 | 6 | 0.91 | 0.16 | 6 | 0.91 | 4 | 0.935 | 8 | 6 | 4 | 6 | 0.39 | 0.23 | 5 | 0.35 |  |
| Self-help with support 6 | 7 | 0.86 | 0.25 | 7 | 0.86 | 7 | 0.930 | 7 | 7 | 7 | 7 | 0.44 | 0.33 | 7 | 2.10 |  |
| Exposure 12 | 8 | 0.85 | 0.28 | 8 | 0.85 | 8 | 0.899 | 5 | 8 | 8 | 8 | 0.45 | 0.35 | 8 | 0.41 |  |
| Anticonvulsants 7 | 9 | 0.81 | 0.27 | 9 | 0.81 | 9 | 0.877 | 13 | 9 | 9 | 9 | 0.49 | 0.32 | 9 | 0.54 |  |
| NSSA 8 | 10 | 0.80 | 0.42 | 10 | 0.79 | 11 | 0.851 | 9 | 10 | 11 | 10 | 0.50 | 0.45 | 10 | 0.61 |  |
| Self-help no support 5 | 11 | 0.75 | 0.25 | 11 | 0.75 | 12 | 0.824 | 16 | 11 | 12 | 11 | 0.55 | 0.33 | 11 | 0.56 |  |
| Psychological placebo 3 | 12 | 0.63 | 0.14 | 12 | 0.63 | 10 | 0.765 | 11 | 13 | 10 | 12 | 0.67 | 0.23 | 12 | 0.71 |  |
| Psychodynamic psychotherapy 14 | 13 | 0.62 | 0.35 | 13 | 0.61 | 13 | 0.641 | 14 | 12 | 13 | 13 | 0.68 | 0.41 | 13 | 1.39 |  |
| Pill placebo 2 | 14 | 0.47 | 0.37 | 14 | 0.45 | 14 | 0.461 | 6 | 14 | 14 | 14 | 0.83 | 0.40 | 14 | 0.95 |  |
| Other psychological therapies 13 | 15 | 0.36 | 0.25 | 15 | 0.35 | 16 | 0.376 | 15 | 16 | 16 | 15 | 0.94 | 0.31 | 15 | 0.68 |  |
| Exercise promotion 4 | 16 | 0.35 | 0.49 | 16 | 0.28 | 15 | 0.280 | 12 | 15 | 15 | 16 | 0.95 | 0.53 | 16 | 1.21 |  |
| Waitlist control 1 | 17 | 0(R) | - | - | 0(R) | - | - | - | - | - | 17 | 1.30 | 0.22 | 17 | 1.46 |  |

R Reference Treatment 1.

***Social Anxiety, Class analysis***

***Type of model****:* Random study effects, random class model: 16 treatment classes based on 40 individual treatments, with waitlist as the reference.^4,5^

***Outcome*:** Standardized Mean Difference

***MCID***: 0.50 ***GRADE probability cutoff****:* 0.975

***EV and LaEV Decision rules***

*Stage 1.* *.* All 16 treatment classes were superior to waitlist control, on both EV and LaEV criteria.

*Stage 2*. A total of 9 treatments were within the MCID of the best treatment on EV and would therefore be recommended, and 6 treatments on LaEV.

***GRADE****:* 4 treatments were promoted to Category 1, those ranked 1,2,6,5 by EV; none are superior to any others by the given criteria, so all are recommended.

***Ranking Systems****:*  Both Pr(V>T) and SUCRA pick out the same 6 top-ranked treatments as EV. Pr(Best) identifies 4 of them among its highest ranked 6.

**Figure S7.** Social Anxiety (Class Analysis)

**
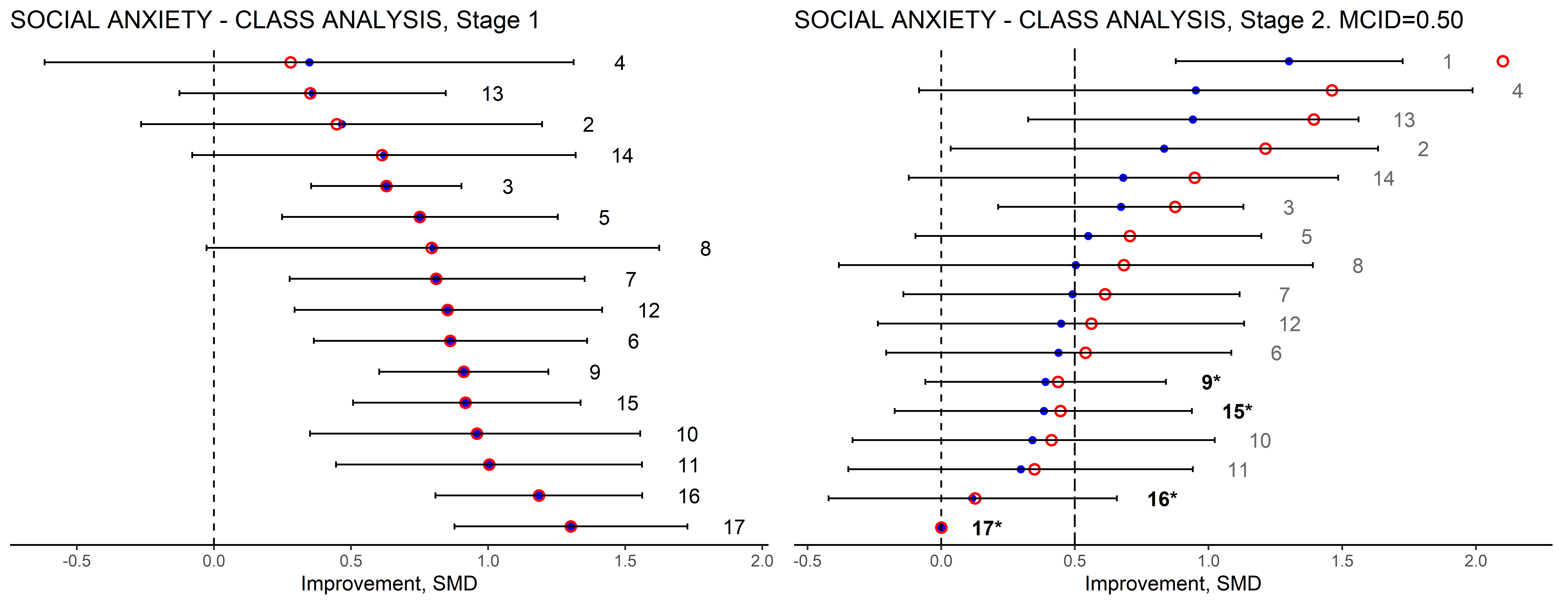
**

**A8. Urinary Incontinence**

T**able S8.** Urinary incontinence. The outcome is probability of achieving continence status, relative to placebo.

| **Treatment**  (numbering as in NICE guidelines) | **STAGE 1**  **Decision Rules** | | | | | | | | **Ranking systems** | | | **STAGE 2**  **Decision Rules** | | | | | **FINAL**  **GRADE**  (0.975)  Category 1 |
| --- | --- | --- | --- | --- | --- | --- | --- | --- | --- | --- | --- | --- | --- | --- | --- | --- | --- |
|  | **EV** | | | **LaEV** | | | **GRADE**  (0.975)  Category 1 | |  |  |  | **EV** | | | **LaEV** | |  |
|  | **Rk** | **EV** | **Sd** | **Rk** | | **LaEV** | **Rk** | **Pr(V>T)** | **P(Best)** | **SUCRA** | **Pr(V>T)** | **Rk** | **EV** | **sd** | **Rk** | **LaEV** |  |
| Oxybutynin IR (1) | 1 | 0.124 | 0.038 | 1 | 0.124 | | 7 | 1.000 | 1 | 1 | 7 | 1 | 0.000 | 0.000 | 1 | 0.000 | 7 |
| Tolterodine IR (4) | 2 | 0.116 | 0.053 | 2 | 0.116 | | 11 | 1.000 | 2 | 2 | 11 | 2 | 0.008 | 0.051 | 2 | 0.020 | 11 |
| Darifenacin (11) | 3 | 0.098 | 0.052 | 3 | 0.098 | | 8 | 1.000 | 4 | 3 | 8 | 3 | 0.026 | 0.071 | 3 | 0.054 | 8 |
| Propiverine IR (5) | 4 | 0.094 | 0.070 | 4 | 0.094 | | 13 | 0.996 | 3 | 4 | 13 | 4 | 0.030 | 0.084 | 4 | 0.066 | 13 |
| Trospium (9) | 5 | 0.084 | 0.047 | 5 | 0.084 | | 6 | 0.993 | 9 | 5 | 6 | 5 | 0.041 | 0.067 | 5 | 0.076 | 6 |
| Oxybutynin ER (3) | 6 | 0.079 | 0.038 | 6 | 0.079 | | 3 | 0.989 | 5 | 6 | 3 | 6 | 0.045 | 0.060 | 6 | 0.081 | 3 |
| Solifenacin (2) | 7 | 0.071 | 0.025 | 7 | 0.071 | | 1 | 0.985 | 6 | 7 | 1 | 7 | 0.053 | 0.053 | 7 | 0.092 | 1 |
| Trospium ER (12) | 8 | 0.070 | 0.028 | 8 | 0.070 | | 10 | 0.982 | 10 | 8 | 10 | 8 | 0.054 | 0.054 | 8 | 0.094 | 10 |
| Oxybutynin TG (13) | 9 | 0.068 | 0.062 | 9 | 0.067 | | 2 | 0.973 | 8 | 9 | 2 | 9 | 0.056 | 0.077 | 9 | 0.105 |  |
| Oxybutynin TD (10) | 10 | 0.064 | 0.031 | 10 | 0.064 | | 5 | 0.970 | 12 | 10 | 5 | 10 | 0.061 | 0.055 | 10 | 0.106 |  |
| Fesoterodine (8) | 11 | 0.057 | 0.020 | 11 | 0.057 | | 12 | 0.953 | 7 | 11 | 12 | 11 | 0.068 | 0.049 | 11 | 0.116 |  |
| Propiverine ER (7) | 12 | 0.055 | 0.029 | 12 | 0.055 | | 4 | 0.901 | 11 | 12 | 4 | 12 | 0.069 | 0.053 | 12 | 0.121 |  |
| Tolterodine ER (6) | 13 | 0.036 | 0.014 | 13 | 0.036 | | 9 | 0.793 | 13 | 13 | 9 | 13 | 0.088 | 0.045 | 13 | 0.153 |  |
| Placebo (14) | - | 0(R) | - | - | 0(R) | | - | - | - | - | - | 14 | 0.124 | 0.038 | 14 | 0.224 |  |

Abbreviations: R Reference treatment 1

***Urinary Incontinence***

***Type of model****:* Fixed study effects, 13 treatments compared to placebo.^7,8^

***Outcome*:**  Probability of improvement in continence status

***MCID***: RR=1.25 ***GRADE probability cutoff****:* 0.975

***EV and LaEV decision rules***

Stage 1. All 13 treatments were superior to placebo on both EV and LaEV criteria

Stage 2. 5 treatments including the best treatment were within the MCID of the best treatment and would be recommended on EV criteria. Only two would be recommended on LaEV.

***GRADE:*** 8 treatments were promoted to Category 1, including 3 of the 4 treatments ranked lowest on EV. As none were superior to any others, all would be recommended.

***Ranking Systems****:*  SUCRA top-ranked the same 5 treatments as EV, Pr(best) 4 of them. Pr(V>T) selected none of them, but privileged treatments 11 and 13 which were estimated with relatively high precision.

**Comments**: The results with LaEV concur with a threshold analysis in which a plausible degree of bias (0.1 on the log odds scale) in the evidence for oxybutynin IR would make tolterodine IR the best treatment.^8^ A further point of interest in this NMA is that the guideline developers made oxybutynin IR (the most effective treatment) the reference treatment, rather than placebo, from the outset. This was because it had been shown to be the best treatment in an earlier evaluation. This suggests that the 2-stage methodology, in which treatments are compared to the best treatment in the second stage, might be readily adopted by clinical decision makers.

**Figure S8** Urinary Incontinence


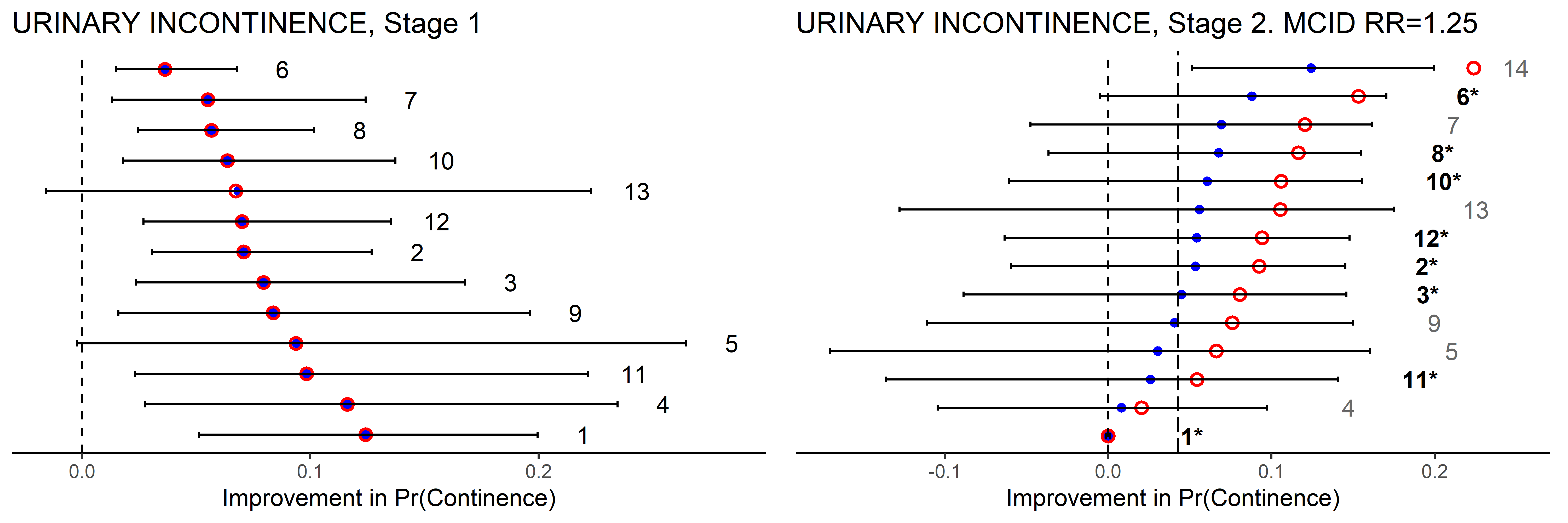


**A9. Tocolytic therapy in Preterm Labour**.^8,9^

**Table S9.** Tocolytic therapy in preterm Labour. Outcome is increase in EGA (weeks)

| **Treatment**  (numbering as in NICE guidelines) | **STAGE 1**  **Decision Rules** | | | | | | | **Ranking systems** | | | **STAGE 2**  **Decision Rules** | | | | | |
| --- | --- | --- | --- | --- | --- | --- | --- | --- | --- | --- | --- | --- | --- | --- | --- | --- |
|  | **EV** | | | **LaEV** | | **GRADE**  (0.975)  Category 1 | |  |  |  | **EV** | | | **LaEV** | | **GRADE**  (0.975)  Category 1 |
|  | **Rk** | **EV** | **Sd** | **Rk** | **LaEV** | **Rk** | **Pr(V>T)** | **P(Best)** | **SUCRA** | **Pr(V>T)** | **Rk** | **EV** | **sd** | **Rk** | **LaEV** |  |
| Prostaglandin inhibs. (2) | 1 | 2.32 | 0.53 | 1 | 2.32 | 1 | 0.992 | 1 | 1 | 1 | 1 | 0.00 | 0.00 | 1(R) | 0.00 | 1 |
| Ca channel blockers (5) | 2 | 1.68 | 0.50 | 2 | 1.68 | 2 | 0.915 | 3 | 2 | 2 | 2 | 0.63 | 0.53 | 2 | 0.71 |  |
| Nitrates (6) | 3 | 1.65 | 0.57 | 3 | 1.65 | 3 | 0.875 | 2 | 3 | 3 | 3 | 0.66 | 0.67 | 3 | 0.79 |  |
| Magnesium sulphate (3) | 4 | 1.28 | 0.50 | 4 | 1.28 | 5 | 0.723 | 6 | 4 | 5 | 4 | 1.03 | 0.50 | 4 | 1.25 |  |
| Betamimetics (4) | 5 | 1.24 | 0.42 | 5 | 1.24 | 4 | 0.716 | 4 | 5 | 4 | 5 | 1.07 | 0.52 | 5 | 1.31 |  |
| Oxytocin RBs (7) | 6 | 0.67 | 1.02 | 6 | 0.52 | 6 | 0.371 | 5 | 6 | 6 | 6 | 1.64 | 1.09 | 6 | 2.47 |  |
| Placebo (1) | 7 | 0(R) | - | - | 0(R) | - | - | - | - | - | 7 | 2.32 | 0.53 | 7 | 3.63 |  |

Abbreviations: inhibs. Inhibitors; Ca Calcium; RBs receptor inhibitors; R Reference Treatment 1

***Tocolytic therapy in preterm Labour***

***Type of model****:* Random study effects, fixed class NMA of 6 treatment classes compared to placebo.

***Outcome*:**  Increase in Expected Gestational Age (EGA) at delivery, measured in weeks.

***MCID***: 1 week ***GRADE probability cutoff****:* 0.975

***EV and LaEV decision rules***

*Stage 1.* All 6 treatments were superior to placebo on both EV and LaEV

*Stage 2.* 3 treatments, including the best treatment, were within the MCID of the best treatment, and would be recommended on both EV and LaEV criteria.

***GRADE****:* A single treatment is promoted to Category 1, and is therefore recommended; the same treatment was top-ranked by EV.

***Ranking systems****:* The three treatments top-ranked on EV and LaEV are also top-ranked by the three probabilistic rankings

**Figure S9**. Tocolytic therapy in preterm Labour


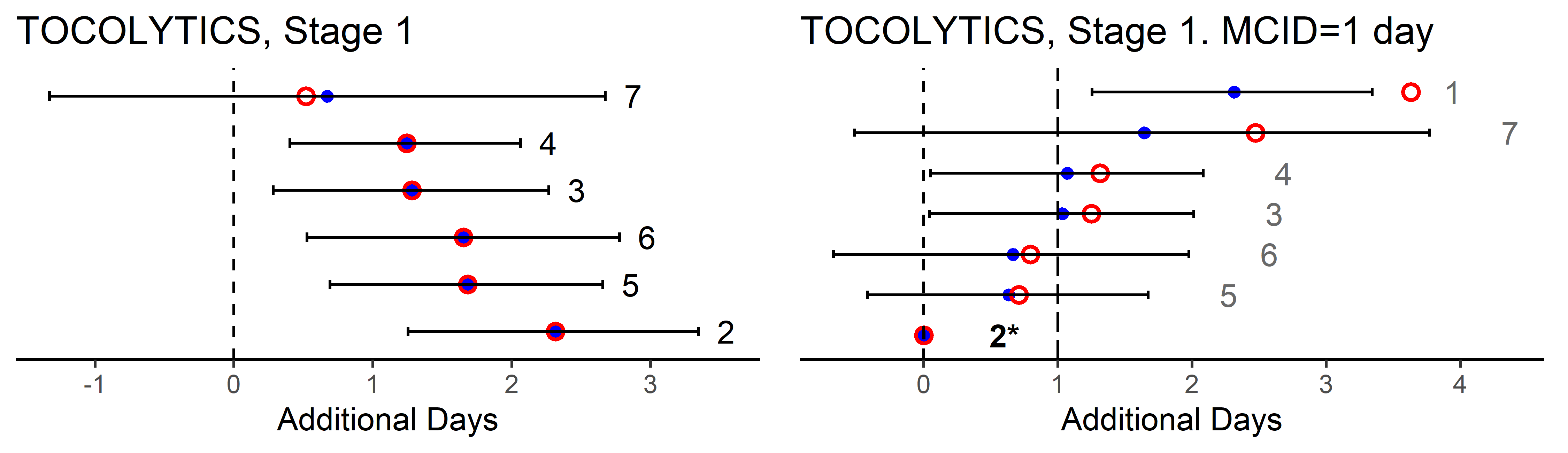


**APPENDIX B: WinBUGS CODE for ILLUSTRATIVE EXAMPLES**

**ILLUSTRATION 1:**

model {

for (s in 1:100) { p[s] <- pow(s/20,-2)

v[s] ~ dnorm(1,p[s])

el[s] <- max(0,-v[s])

laev[s] <- 1-el[s]

ev[s] <- 1.0

pr[1,s] <- step(v[s]-0)

pr[2,s] <- step(v[s]-0.5)

pr[3,s] <- step(v[s]-1.0)

pr[4,s] <- step(v[s]-1.5)

pr[5,s] <- step(v[s]-2.0)

pr[6,s] <- step(v[s]-3.0) }

}

**ILLUSTRATION 2:**

model {

for (s in 1:3) { p[s] <- pow(sd[s],-2)

v[s] ~ dnorm(m[s],p[s])

el[s] <- max(0,-v[s])

laev[s] <- v[s]-el[s] }

}

list(m=c(1,2,3), sd=c(0.1,1,1.5))

**ILLUSTRATION 4:**

model {

for (i in 1:nm) { for (j in 1:nsd) {

m[i,j] <- 1+i/10 # generates EVs at 1.1, 1.2, 1.3, 1.4, 1.5

sd[i,j] <- sdlist[j]

pr[i,j] <- pow(sd[i,j],-2)

t[i,j] ~ dnorm(m[i,j],pr[i,j])

ev[i,j] <- t[i,j] # incremental benefit relative to standard

el[i,j] <- max(0,-ev[i,j]) # exp incr value if perfect info

laev[i,j] <- ev[i,j] - el[i,j] # loss-adjusted expected value

pv[1,i,j] <- step(t[i,j]-0.6)

pv[2,i,j] <- step(t[i,j]- 1.3)

pv[3,i,j] <- step(t[i,j]-2.0)

tx[(i-1)*nsd + j] <- t[i,j] }}

for (i in 1:25) { rk[i] <- 26 - rank(tx[],i) # mean rank (check)

pb[i] <- equals(rk[i],1) # probability best

for (j in 1:25) { pbij[i,j] <- step(tx[i]-tx[j]) - equals(i,j) } # count if (i > j)

su[i] <- sum(pbij[i,]) / 24 } # SUCRA

}

list(nm=5, nsd=5, sdlist=c(1,2,3,4,5) )

**APPENDIX C: WINBUGS CODE FOR NMAs**

**Additional code for Social anxiety Class analysis, 16 treatments and placebo**

**# LaEV CODE ****************************************************************************

# MCID: 0.5 SMD units

for (k in 1:16) { f[k] <- -m[k+1] # EV-1, reduction depression score rel to placebo

la[k] <- f[k] - max(0,-f[k]) # LaEV-1

rnk[k] <- 17 - rank(f[],k) # SUCRA

bst[k] <- equals(rnk[k],1) # Pr(Best)

pv[k] <- step(f[k] - 0.50) } # Pr(V>T)

# Treatment m[17] is best

for (k in 1:17) { f2[k] <- -m[17] + m[k] # EV-2

la2[k] <- f2[k] + max(0,f2[k]-0.50) } # LaEV-2

# GRADE: Look at probability that 17x17 differences between effects exceed the threshold.

for (i in 1:16) {for (j in i+1:17) { gr[j,i] <- step(-m[j]+m[i] - 0.50 ) }}

for (j in 1:16) {for (i in j+1:17) { gr[j,i] <- step(-m[j]+m[i] - 0.50) }}

**WINBUGS System files for all 10 NMAs are available** [***here***](https://www.bristol.ac.uk/population-health-sciences/centres/beam-centre/mpes/nice/reportsandpublications.html)**. They include the original code used for the NMA analysis, with the additional code for LaEV, GRADE and rankings, along with the data and initial values.**

**Supplementary Material References**

1. National Institute for Health and Care Excellence. Acne vulgaris: management. NICE Guideline [NG 198]. London, 2021.

2. National Institute for Health and Care Excellence. Depression in adults: treatment and management. NICE Guideline [NG 222]. London, 2022.

3. National Institute for Health and Clinical Excellence. Joint replacement (primary): hip, knee and shoulder. Network meta-analysis and cost analysis of methods for tranexamic acid administration. NG157. London, 2020.

4. National Institute for Health and Clinical Excellence. Social Anxiety Disorder: Recognition, Assessment and Treatment. CG159. London, 2020.

5. Phillippo DM, Dias S, Welton NJ, Caldwell DC, Taske N, Ades AE. Threshold Analysis as an Alternative to GRADE for Assessing Confidence in Guideline Recommendations Based on Network Meta-analyses. *Ann Intern Med* 2019; **170**: 538-46.

6. Mayo-Wilson E, Dias S, Mavranezouli I, et al. Psychological and pharmacological interventions for social anxiety disorder in adults: a systematic review and network meta-analysis. *Lancet Psychiatry* 2014; **1**: 368-76.

7. National Institute for Health and Clinical Excellence. Urinary incontinence in women: the management of urinary incontinence in women. London, 2013.

8. Phillippo DM, Dias S, Welton NJ, Ades AE. Threshold Analysis in NICE Guideline Development, [https://www.bristol.ac.uk/population-health-sciences/centres/cresyda/mpes/nice/tsu-reports/](https://url.avanan.click/v2/r02/___https://www.bristol.ac.uk/population-health-sciences/centres/cresyda/mpes/nice/tsu-reports/___.YXAxZTpjYW1icmlkZ2Vvcmc6YTpvOjViMTU0NzMyYjRjNDM1ZjliYmEwNTY3OWJjYWMxNWFlOjc6NjI1MTowMjUxNjI5MmIzYzgzMjJiNmNiZDAwY2RlNjIzNTA1OGY3MTE5M2E3ZDU2YmM0OTIyMjkyOTFiZmM4ZDc0Nzk2OnA6VDpG), 2016.

9. National Institute for Health and Clinical Excellence. Preterm labour and birth. London, 2015.
